# Lightweight neural networks with anti-colored noise for bearing fault diagnosis using deep separable convolution and transfer learning

**DOI:** 10.1038/s41598-025-28332-8

**Published:** 2025-12-29

**Authors:** Jiping Li, Nan Wang, Dan Wang, Guowei Gao, Wujiu Pan

**Affiliations:** 1 School of Mechanical Engineering, Shenyang Urban Construction University, Shenyang, 110167 People’s Republic of China; 2 Department of Fundamental Science, Army Artillery Air Defense Academy Sergeant School, 110867 Shenyang, People’s Republic of China; 3 School of Mechatronics Engineering, Shenyang Aerospace University, Shenyang, 110136 People’s Republic of China

**Keywords:** Fault diagnosis modeling, Lightweight neural networks, Transfer learning, Variable adaptive characteristics, Deep separable convolution, Engineering, Mathematics and computing

## Abstract

Practical bearing fault diagnosis faces several key challenges: noise interference, performance degradation under varying operating conditions, and slow diagnostic speed. For such engineering problems, this article combines deeply separable convolution to establish three lightweight neural network fault diagnosis models—MobileNet-DLCNN, ShuffleNet-DLCNN and SqueezeNet-DLCNN. At the same time, the failure mechanism of rolling bearings is studied. This article incorporates colored noise into the standard bearing failure datasets from Case Western Reserve University (CWRU) and the American Society for Machinery Failure Prevention Technology (MFPT), and conducts noise resistance training under variable adaptive characteristics, compares the diagnostic ability with the datasets before adding colored noise. Then, the identification and classification abilities of the three lightweight convolution models proposed under each dataset, as well as the transfer learning adaptive ability of the three models between different datasets and working conditions, are verified through comparative experiments. The experimental results show that SqueezeNet-DLCNN has the best diagnostic performance among the three models, and it can complete the recognition and classification of all data in about one minute with an accuracy of 97%. The lightweight convolution designed in this article has the advantages of strong noise resistance, high efficiency, and fast speed.

## Introduction

With the continuous development of industrial technology, the volume of rotating machinery continues to increase and its structure becomes increasingly complex. Rolling bearing is one of the most prone components to malfunction in rotating machinery equipment that operates in high load and fast speed environments. Therefore, early fault diagnosis of rolling bearings is crucial. The nonlinear characteristics of bearings have a significant impact on the vibration characteristics of rotor systems^1–3^. In engineering practice, there are three most common and typical failure forms of rolling bearings, namely pitting, wear, and gluing. The time from the occurrence of gluing to the entire bearing being damaged is very short, so it is difficult to diagnose it in a timely manner through routine inspections.

During the long-term continuous operation of rolling bearings, there is no significant change in the vibration signal characteristics under stable conditions. However, as the operating time increases, abnormal situations such as wear, corrosion, and damage will occur, the width of the vibration signal will gradually increase^4–6^. The traditional fault diagnosis method mainly extracts from the time-domain and frequency-domain characteristics of vibration signals, and then judges whether the working condition has deteriorated based on the vibration characteristics to determine whether a fault has occurred. However, due to the noise generated by machine operation, the measured data may not be standardized. In response to the above situation, Sun et al.^7^ proposed a new method for bearing fault diagnosis based on empirical mode decomposition (EMD) and improved Chebyshev distance, which has good robustness and accuracy. Bastami et al.^8^ used a spectral kurtosis method based on optimized pulse response wavelet for bearing fault diagnosis, using a special wavelet transform to obtain the SK image. Machine learning and deep learning technology have a wide range of applications in data processing, which can fully mine effective information of rolling bearings and diagnose them correctly. Based on the improvement of the multi-scale convolutional neural network bearing fault diagnosis model, the feature attention mechanism was added by Xu et al.^9^ to establish a new end-to-end diagnostic model with good extrapolation performance. Han et al.^10^ used hybrid distance guided adversarial networks to extract diverse feature information and achieve intelligent fault diagnosis under different operating conditions. With the development of the times, neural network technology is becoming increasingly mature. Due to its efficiency and convenience, scholars have focused on the field of combining neural networks with fault diagnosis.

Neural networks can effectively solve classification problems, helping to group unlabeled data based on the similarity between sample inputs^11^. As of now, various neural network models have been proposed by the academic community, Such as Feedforward Neural Networks(FNN)^12^, Convolutional Neural Network(CNN)^13,14^, Recurrent Neural Network(RNN)^15^, Artificial neural networks(ANN)^16^, etc. When there is a shortage of training samples, people begin to focus on reducing the complexity of the model.

Compared to processing the trained model, the design of lightweight models is a completely new approach. The core of this method is to design a more effective convolutional method to reduce network parameters without affecting network performance as much as possible, and to lightweight transform the network from both volume and speed while maintaining accuracy as much as possible. The lightweight model has fewer network parameters and faster calculation speed.

However, despite their fast speed, lightweight models exhibit limited capability in diagnosing faults across different operating conditions. Transfer learning refers to the process of adapting a model trained on one task to a related but different task, often with minimal additional training data. In other words, it is the influence of one type of learning on another, or the influence of acquired experience on completing other activities. In the field of intelligent fault diagnosis, transfer learning has made many outstanding achievements. But in practical applications, due to the complexity and changes in the working environment, stable data cannot be received. Ignoring this issue will greatly reduce the accuracy of diagnosis. As an important branch of deep learning, transfer learning has been widely studied by machine learning scholars in the past few years^17,18^. Owing to the fact that many practical applications in industrial scenarios do not allow equipment to operate under fault conditions for a long time, obtaining sufficient fault data is very difficult. Wan et al.^19^ proposed a fault diagnosis model for bearings with unlabeled data based on transfer learning based on hierarchical decoding and attention mechanism to ensure that the characteristics of source domain and target domain belong to the same fault type.

The energy propagation graph neural network proposed by Li et al.^20^ effectively enhances the model’s analytical ability on out-of-distribution fault samples by explicitly modeling the energy propagation paths between nodes, providing a new approach for handling unknown operating conditions. At the same time, in the face of the challenge of multi-source heterogeneous sensor data fusion, theory-driven fusion strategies demonstrate great potential. Li et al.^21^ proposed a multi-channel fusion framework (GDSI) based on the generalization error bound theory. It provides an interpretable theoretical foundation and optimization objective for the dynamic and robust fusion of multi-source signals through covariance constraints and dual-metric trust mechanisms, significantly improving the stability of the diagnosis system under noise interference and distribution changes. These works mark the development of fault diagnosis research towards a deep integration of theoretical rigor, lightweight architecture, and environmental robustness.

At present, most of the models used in transfer learning are complex, and it takes a long time to process the data. It may also be impossible to calculate due to too much calculation or too deep convolution layer. Thus, three lightweight anti colored noise neural network models are proposed in this paper. These models consider the integration of transfer learning and deeply separable convolution, which can significantly improve the diagnostic ability under noisy and cross operating conditions. The main contributions of this article include: (1) Starting from the actual working conditions, lightweight neural network models based on MobileNet, ShuffleNet, and SqueezeNet were constructed, namely Mobile-DLCNN, Shuffle-DLCNN, and Squeeze-DLCNN. (2) Applying deep separable convolution to extract feature parameters reduces both the number of parameters and computational costs compared to conventional convolution operations. (3) Considering the actual noise environment in industrial scenarios, the three models proposed in this article have good anti-interference ability. (4) Based on the established model, multiple control experiments are conducted to demonstrate that the three models established in this paper exhibit excellent diagnostic speed and generalization characteristics.

This article is divided into five parts. After the introduction, the second section introduces transfer learning and the selection of lightweight neural networks. The third section elaborates on the construction process of three fault diagnosis models in detail. In the fourth section, the model constructed in the third section was used to conduct a control experiment with multiple sets of different variables. The fifth section provides the conclusion.

## Application technology and data preprocessing of fault diagnosis model theory

### Overview of transfer learning

**Fig. 1:**
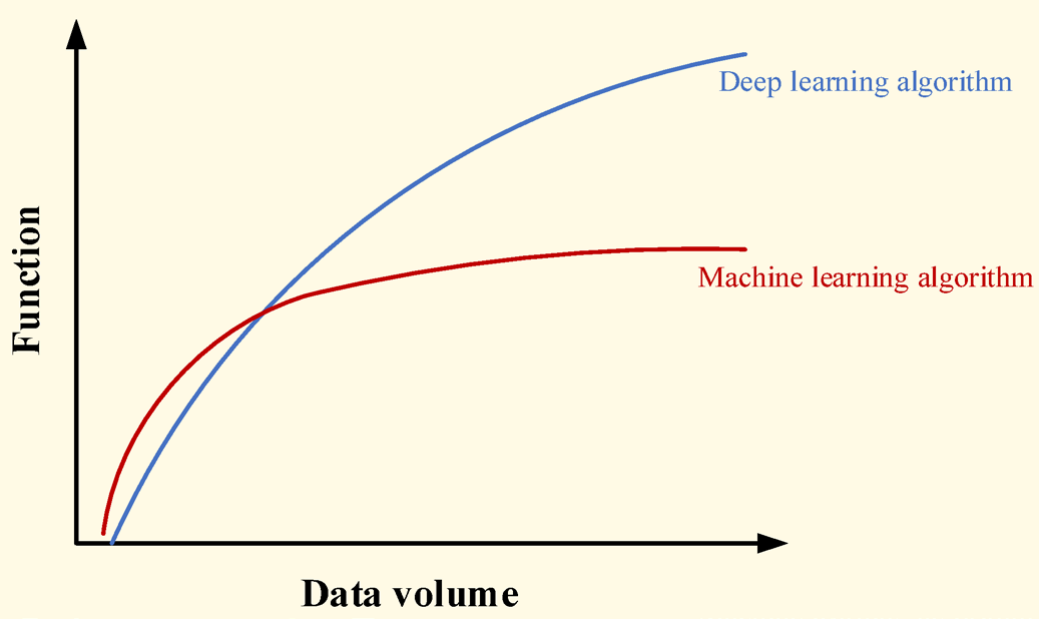
Comparison of deep learning and machine learning performance.

Machine learning is the process of predicting and judging other data by learning computer data. In other words, the computer uses the acquired data to establish a model, and then uses this model to predict. This process is somewhat similar to the learning process of humans, for example, after gaining certain experience, people can predict new problems. Machine learning can be divided into supervised learning, semi supervised learning, unsupervised learning and more complex reinforcement learning. Despite recent attention to machine learning, the clear elucidation of causal relationships in industrial machine learning models remains an open topic that requires further research. Deep learning is a subset of machine learning that uses neural networks with many layers (deep architectures) to model complex patterns in large amounts of data. It is often characterized as a ‘black box’ due to the complexity and lack of interpretability of its internal processes. Machine learning is the methodology of deep learning, and mathematics is the theoretical support behind it. Due to the ever-changing nature of training data, using machine learning methods may find it difficult to process unstructured data. Deep learning models can comprehensively analyze unstructured data without the need for manual feature extraction. The application of deep learning can conduct deeper analysis of massive data and provide new insights. The most important difference between deep learning and traditional machine learning will manifest as the size of the data increases. As shown in Fig. 1, in the case of limited data, deep learning algorithms may encounter problems. This is because deep learning algorithms require massive amounts of data to fully understand it. In this context, traditional machine learning algorithms and manually generated rules have become advantages.

With the continuous deepening of artificial intelligence and machine learning, the principles, algorithms, models, etc. of learning have been greatly developed, and a large number of research results have also emerged. Transfer learning is a kind of research which is widely used in computer vision and natural language processing. Traditional machine learning only works well when both the training and test set data come from the same feature space and are uniformly distributed, and the model needs to be retrained every time the data is changed. In machine learning, transfer learning can use the similarity of data, tasks or models to apply the previously learned models and knowledge to new fields.

### Lightweight convolution

In practical applications, convolutional computation is generally in millisecond units. To meet practical usage requirements, it is necessary to improve processor performance or reduce computational complexity. From this, lightweight convolution has been proposed, which can be understood as a network compression method aimed at reducing computational complexity. This section provides a brief introduction to the three lightweight models proposed in recent years, which are: MobileNet, ShuffleNet, SqueezeNet.

**Fig. 2:**
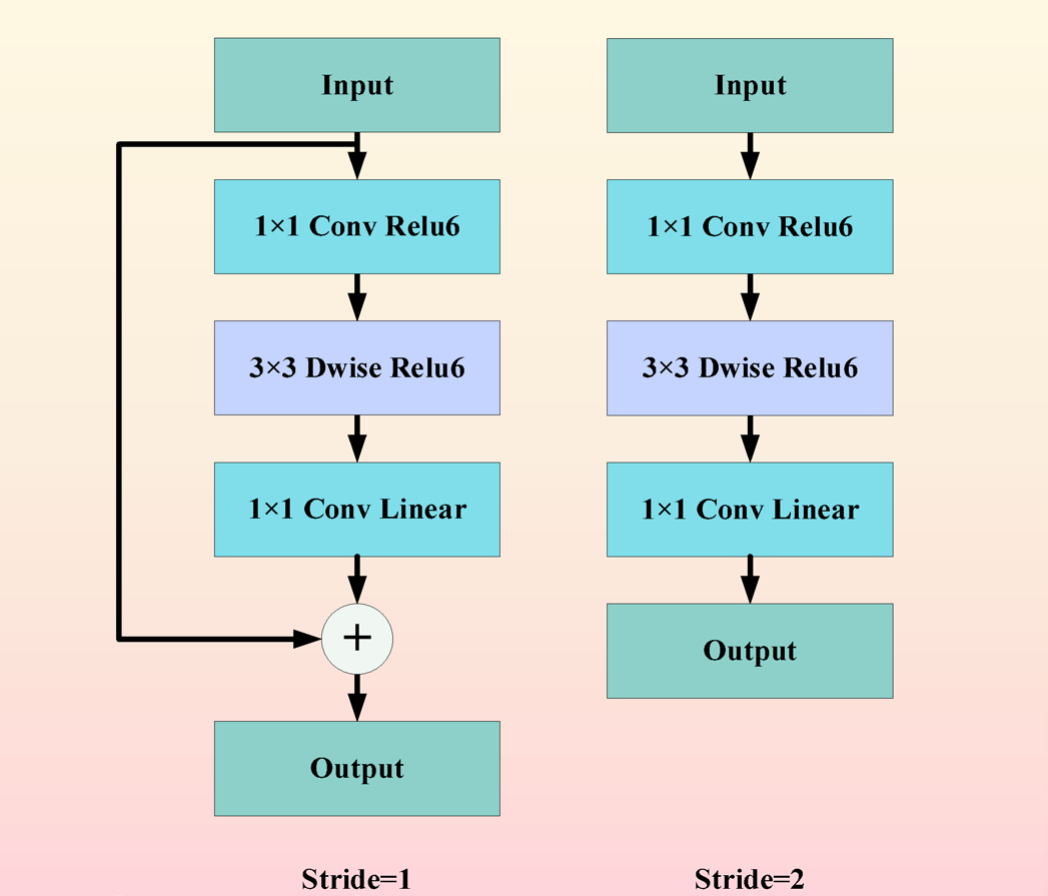
MobileNet flowchart.

Compared to regular convolutions, the MobileNet model has fewer parameters and faster computation speed. The MobileNet model is based on deep separable convolution, which is a form of decomposed convolution. This form decomposes the standard convolution into a depth wise convolution and a called point wise convolution that 1 × 1 convolution. As shown in Fig. 2.

**Fig. 3:**
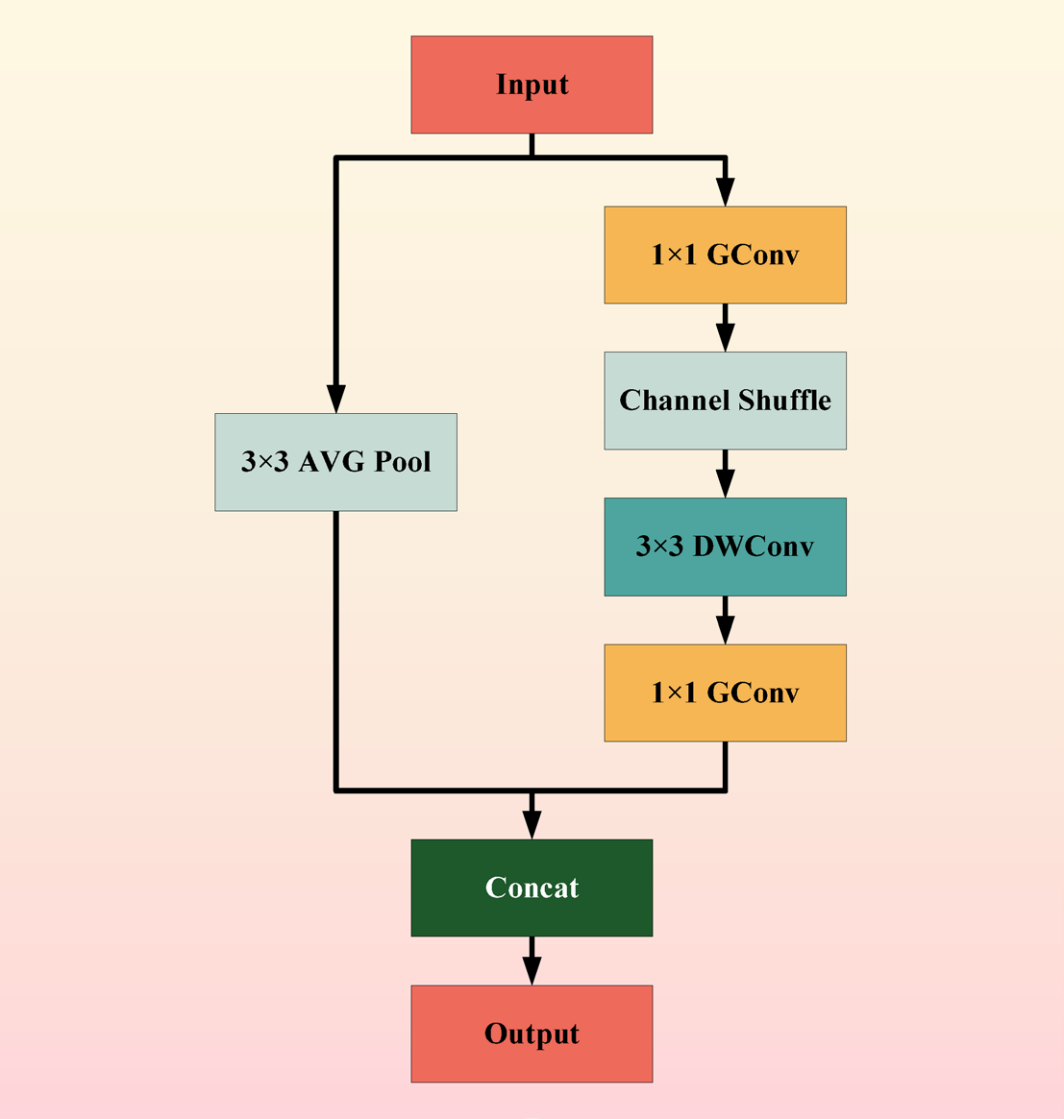
ShuffleNet flowchart.

Two innovative designs were used in the ShuffleNet model: pointwise group convolution and channle shuffle. Point by point group convolution reduces the computational complexity of point by point convolution, and channel shuffling improves the information flow across feature channels. The proposal of these two innovative points enables the ShuffleNet network to maintain accuracy while greatly reducing computational costs. As shown in Fig. 3.

**Fig. 4:**
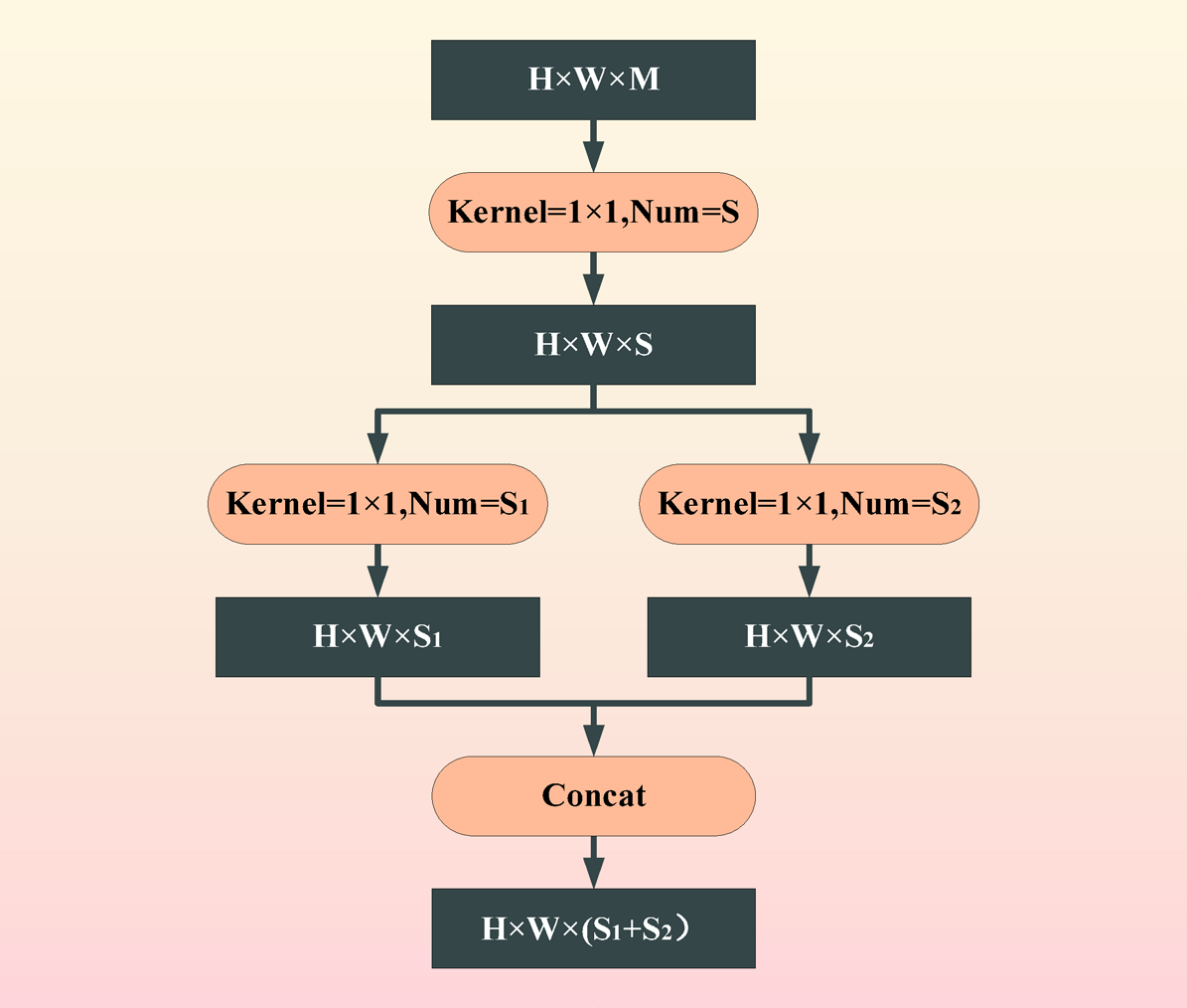
SqueezeNet flowchart.

The SqueezeNet model was proposed by Forrest et al. in 2016. Its innovation is to add a squeeze layer to the convolution, which adopts 1 × 1. The main purpose of convolution is to reduce the dimensionality of the feature map in the previous layer^22^. SqueezeNet series reduced by 3 × 3. The number of input channels for convolutional kernels and the use of delayed down sampling belong to a refined and simplified lightweight convolutional neural network structure. As shown in Fig. 4.

### Deeply separable convolution

Convolution is a widely used algorithm in signal processing, image recognition, and other engineering technologies. Separable convolutions include Spatially Separable Convolution (SSC) and Depthwise Separable Convolution (DSC). SSC divides the convolution of n × n into 1 × n and n × 1 to calculate. It is rarely used in general. DSC is an algorithm derived from the improvement of standard convolution in convolutional neural networks, which reduces the number of parameters required for convolutional calculations by splitting the correlation between spatial dimensions and channel depths. In short, deep separable convolution is the process of splitting a regular convolution into two parts, the first to reduce model parameters and the second to deepen model channels. As a substitute for ordinary convolution, DSC’s biggest advantage is its high computational efficiency. Building lightweight models using DSC is a very common practice nowadays.

**Fig. 5:**
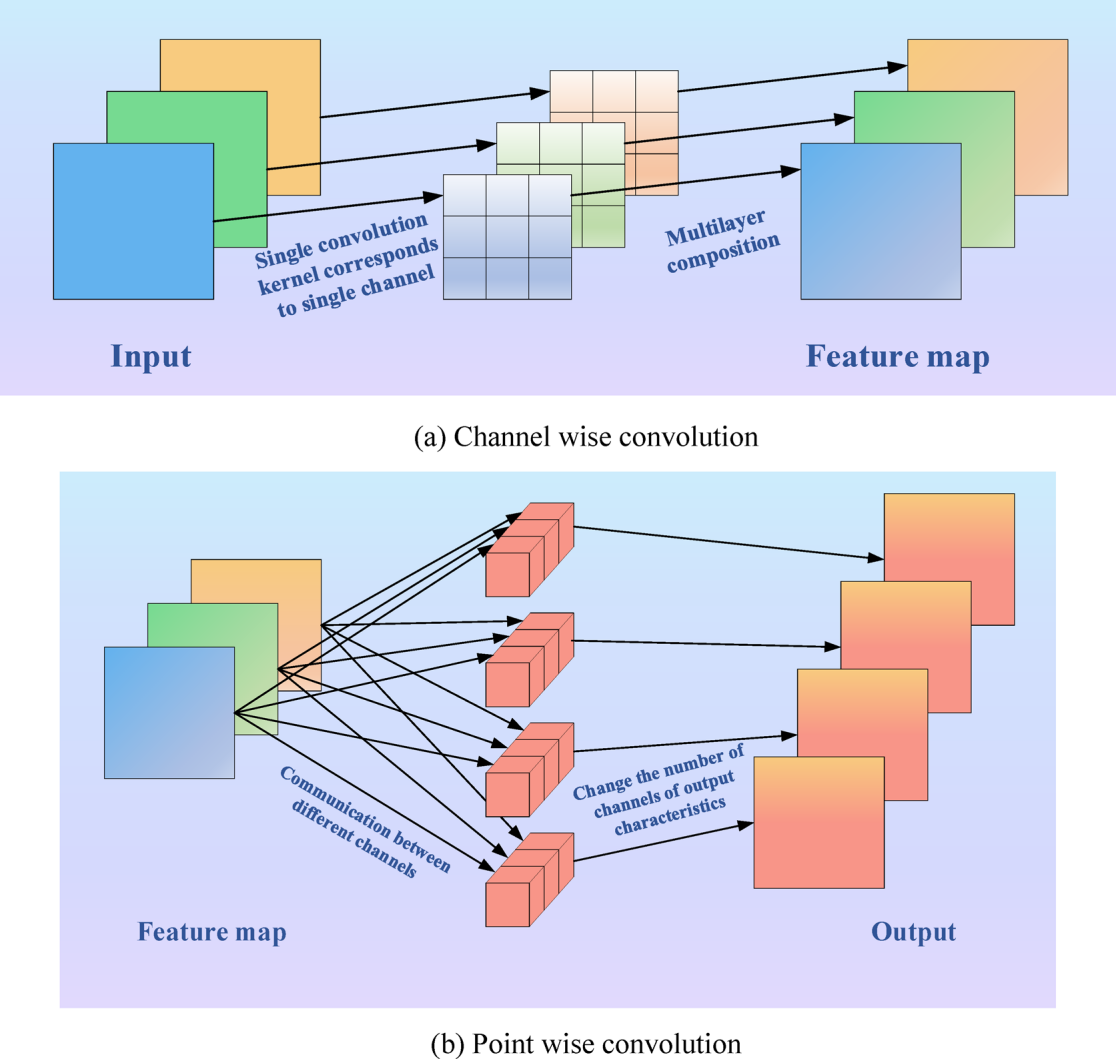
Schematic diagram of deeply separable convolution.

Deep separable convolution is mainly divided into channel wise convolution and point wise convolution. Each channel is convolved into a convolutional kernel responsible for each channel, and the final features are obtained by stacking. Point wise convolution is the execution of 1 × 1 convolution for feature extraction on a single point. As shown in Fig. 5.

### Fault mechanism of rolling bearings

Rolling bearings are the function of changing the distance between bearings in a machine to ensure relative motion of the machine. Using bearings in this way can save more energy in machine production, while using bearings can make machine production more stable. In engineering, there are three most common and typical failure forms of rolling bearings, namely pitting, wear, and bonding. For existing standard bearing datasets, faults are usually manually imposed and can be divided into outer ring faults, inner ring faults, rolling element faults, and cage faults. The expression for a fault can be written as

1 $$\Delta = \sum\limits_{k = - \infty }^{ + \infty } {d\delta (t - kT)}$$

where, *d* is the intensity of the impact pulse, *δ* Is the unit impulse function, *T* is the pulse interval, and *k* is taken as an integer.

Rolling bearings usually only generate vibration in the event of a malfunction, and there are many factors that can cause rolling bearing vibration, but the main factors can be classified into three aspects. Firstly, the vibration of rolling bearings caused by increased production friction during operation is the most common vibration and is common in rolling bearings that have been in operation for a long time. Secondly, the internal degradation of rolling bearings leads to vibration of rolling bearings, which is generally more obvious when rolling bearings fail. Finally, improper use of the rolling bearing under normal working conditions can cause vibration.

### Data preprocessing

#### Case Western Reserve University standard bearing failure dataset

For experimental data, this article uses the Case Western Reserve University standard bearing failure dataset (CWRU)^23^. There are three main types of faults in bearings, namely the inner race, outer race, and ball. Each type of fault diameter is divided into 0.007 inches, 0.014 inches, and 0.021 inches. This forms nine types of faults and one normal type of data. Since the accelerometer is used to collect vibration data, the accelerometer will be placed on the drive end (DE), fan end (FE) and base (BA) of the motor housing, so there will be data measured at three different sensor positions. The specific parameters are shown in Table 1.

**Table 1:** CWRU fault type parameter.

Fault type	Fault diameter /inches	Label
Ball	0.007	1
	0.014	2
	0.021	3
Outer race	0.007	4
	0.014	5
	0.021	6
Inner race	0.007	7
	0.014	8
	0.021	9
Normal	0	10

#### American society for machinery failure prevention technology standard bearing failure dataset

The MFPT bearing data set is also used to verify the proposed model. It includes 17 sets of fault conditions and 3 sets of normal data. The load of the normal bearing condition is 270 lbs, and the input shaft speed is 25 Hz; Three outer rings failure load 270 lbs, input shaft speed 25 Hz; The load of 7 outer ring failures is 25, 50, 100, 150, 200, 250, 300 lbs respectively, and the input shaft speed is 25 Hz; The load of the seven inner ring failures is 0, 50, 100, 150, 200, 250, 300 lbs respectively, and the input shaft speed is 25 Hz.

The MFPT file is in mat format, and each mat file contains four datasets, namely sr, gs, load, and rate. sr is the sampling frequency, gs is the fault data to be extracted, load is the load size, and rate is the speed size. In order to unify with the bearing fault dataset of Case Western Reserve University, nine fault scenarios and one normal scenario were selected in this article to form a dataset for testing. The specific parameters are shown in Table 2.

**Table 2:** MFPT fault type parameter.

Fault type	Load/lbs	Sampling frequency	Label
Outer race	270	97,656	1
	25	48,828	2
	50	48,828	3
Ball	100	48,828	4
	150	48,828	5
	200	48,828	6
Inner race	50	48,828	7
	100	48,828	8
	150	48,828	9
Normal	270	97,656	10

#### Dataset augmentation

**Fig. 6:**
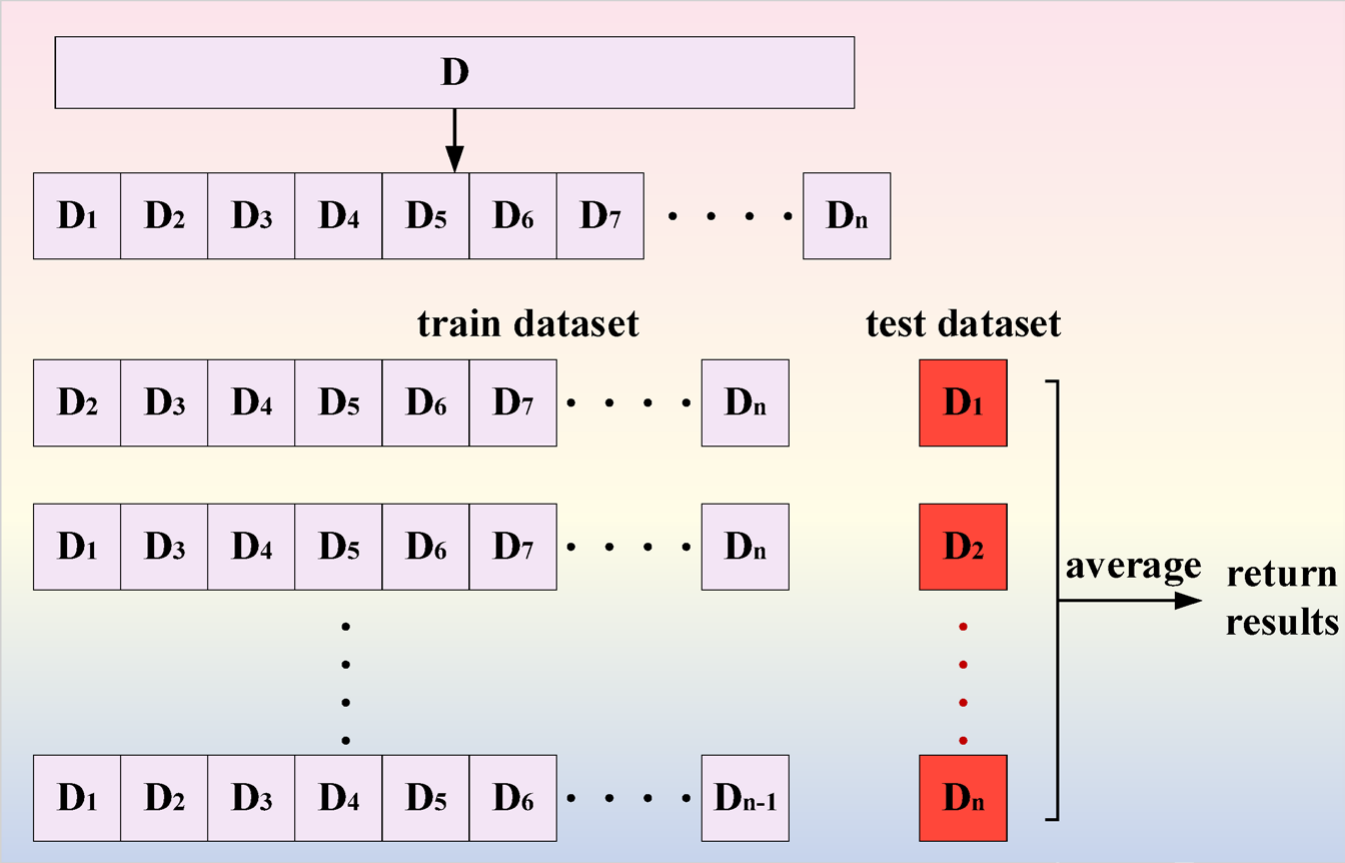
Schematic diagram of cross validation set method.

Cross validation set refers to splitting the dataset into k mutually exclusive subsets. In order to ensure data consistency, the subsets are obtained through stratified sampling. During training, the k-1 subset is used as the training set, and the remaining subset is used as the test set. As shown in Fig. 6.

The goal of data augmentation is to make limited data have comparable value to more data, without significantly increasing the data. In terms of data density, reducing the number of majority class data, eliminating overlap, and removing noise can achieve a balanced dataset. Cross validation can be used when data samples are limited^24–26^. Before constructing the sample, it is necessary to normalize the original dataset so that the data d^*^ is between [0, 1], as shown in Eq. (2).

2 $$d^{*} = \frac{{d - {\mathrm{Min}}(D)}}{{{\mathrm{Max}}(D) - {\mathrm{Min}}(D)}}$$

where *D* represents the original time series signal; *d* represents a point in *D*.

#### Color noise simulation

Rolling bearings themselves usually do not produce noise, and the so-called bearing noise is actually the sound emitted by the surrounding structure of the bearing when it vibrates directly or indirectly. Therefore, noise issues should usually be considered and addressed as vibration issues related to the overall bearing. Vibration and noise often coexist, and the root cause of noise can be attributed to vibration. Therefore, solving noise problems should start with reducing vibration. Under axial load, there is a slight change in the number of loads applied to the rolling element during rotation, causing the bearing to deviate slightly in the direction of the load, resulting in irresistible vibration^27–29^.

White noise is a kind of noise with equal noise energy in each band of equal bandwidth in a wide frequency range. Signal-to-noise ratio is the difference between a normal sound signal and a noise signal without a signal, represented by *snr*. The signal-to-noise ratio is a ratio and the formula for defining signal-to-noise ratio is

3 $$snr = 10\lg \frac{{P_{S} }}{{P_{n} }}$$

where *P*_*S*_ is the signal power, *P*_*n*_ is the noise power.

However, due to the idealization of white noise, the noise in industrial sites is much more complex than white noise. Therefore, we chose colored noise for simulation in the article. Colored noise refers to the noise with uneven power Spectral density function. Colored noise is generated by applying a specific filter to white noise, where the filter’s frequency response determines the power spectral density of the noise. The shape of this spectral density function defines the ‘color’ of the noise, which can be tailored to mimic real-world industrial noise conditions. White light contains all colors, so the characteristic of white noise is that it contains various types of noise.

**Fig. 7:**
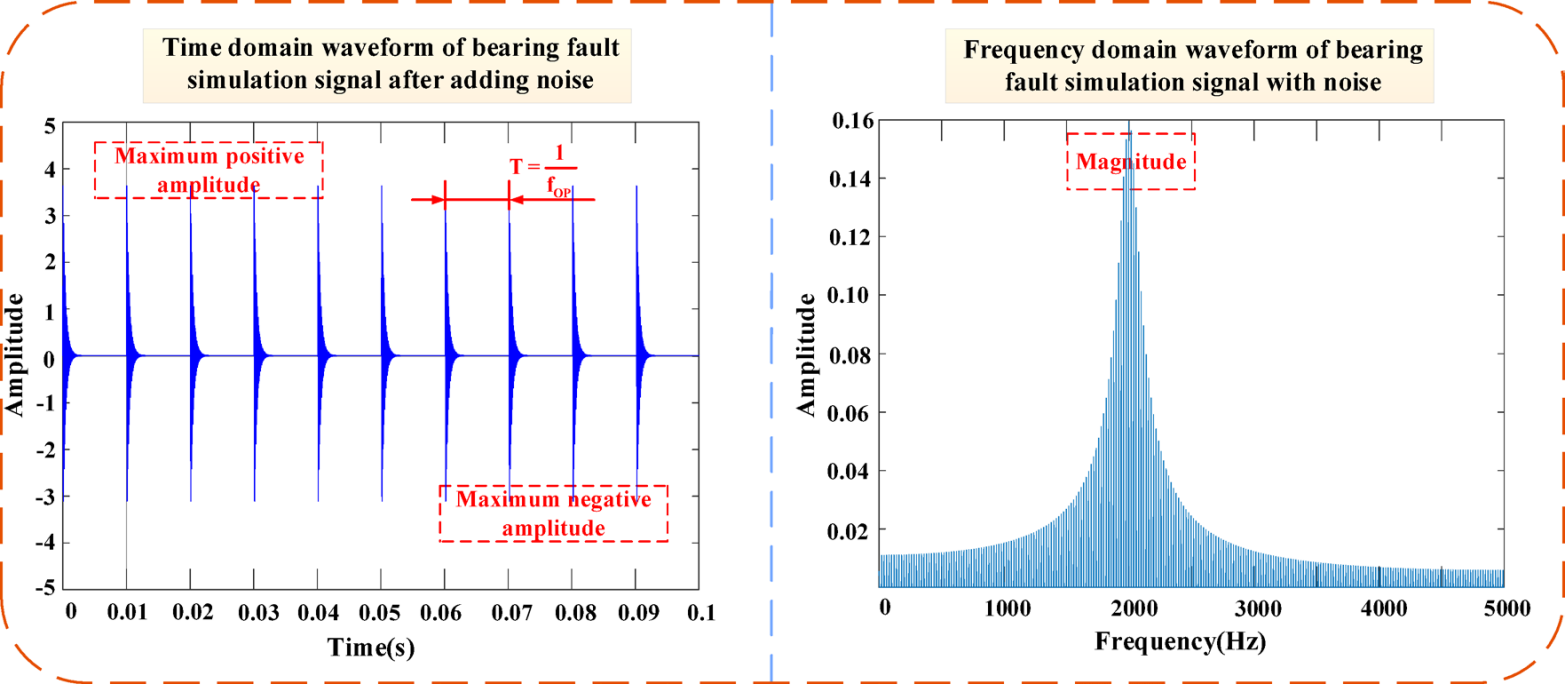
The impact of changes in signal-to-noise ratio on bearing signals. (**a**) Vibration signal at snr = 5. (**b**) Vibration signal at snr = 15.

**Fig. 8:**
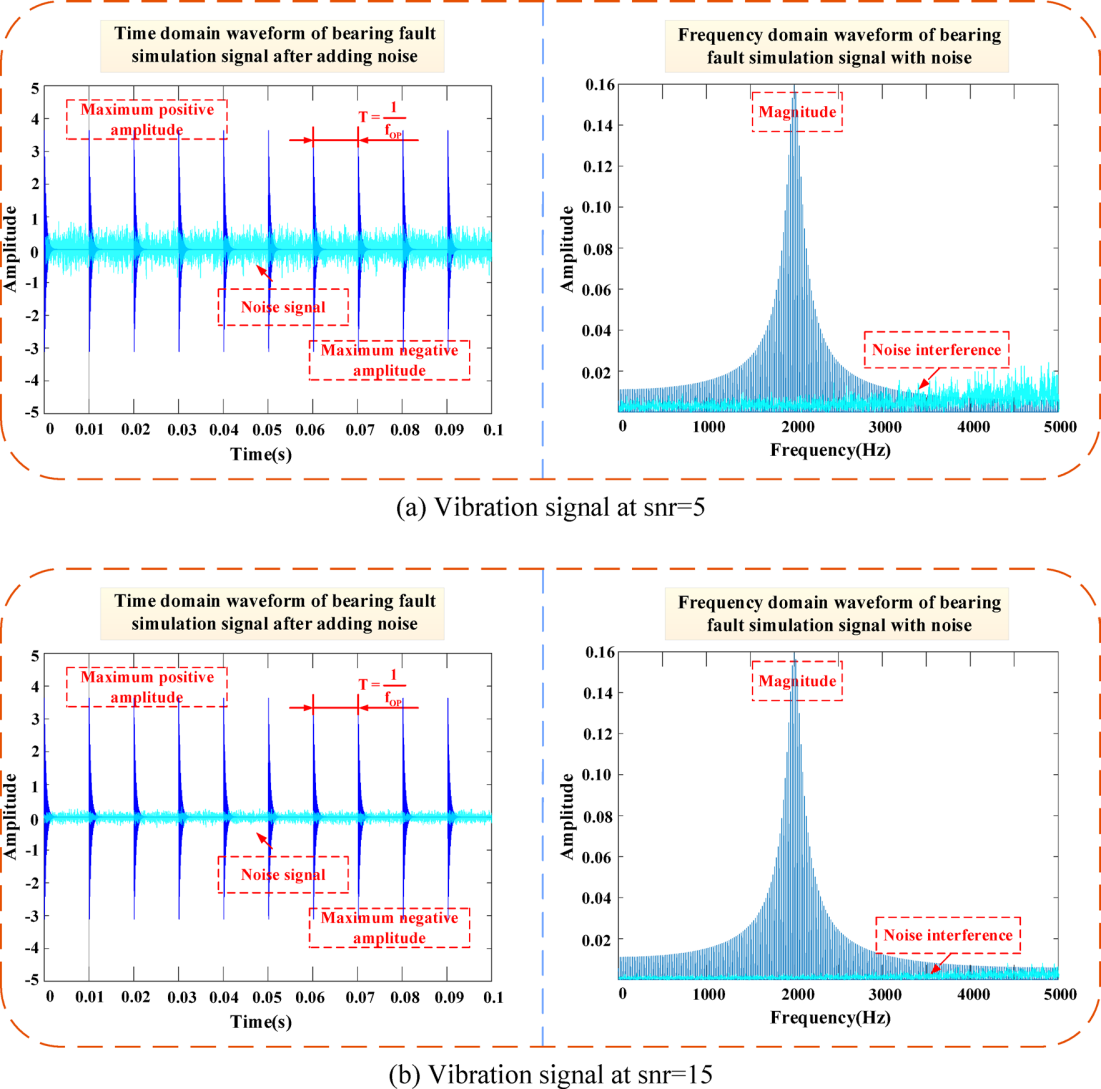
Normal vibration signal.

**Fig. 9:**
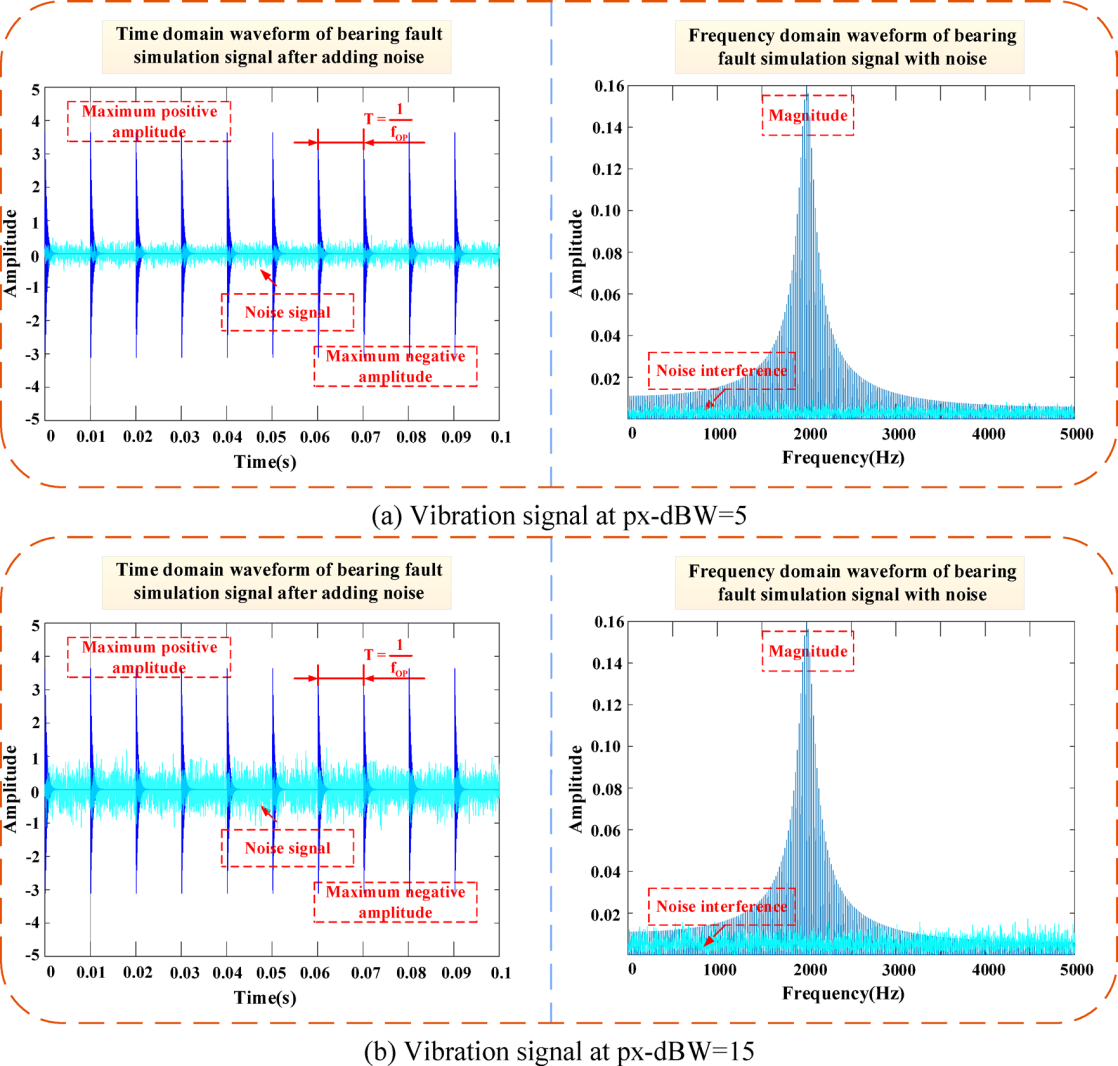
The impact of changes in specified power on bearing signals. (**a**) Vibration signal at px-dBW = 5. (**b**) Vibration signal at px-dBW = 15.

In communication and data measurement, we hope that the signal-to-noise ratio (SNR) is as high as possible. As can be seen from Fig. 7, as the SNR increases, the bearing vibration signal tends to stabilize. Furthermore, Fig. 8 shows the morphology of normal vibration signals, while Fig. 9 illustrates the impact of different specified powers (px-dBW) on the bearing signals.

With the signal-to-noise ratio snr and the specified power px-dBW of the signal, the power of the Gaussian white noise to be added can be calculated. Further filtering can generate colored noise.

## Fault diagnosis model

### The construction of lightweight convolutional modules

With the continuous development of lightweight neural networks, more and more design methods have been proposed and have been successfully used to solve various problems. The design of lightweight convolution reduces the number of convolutional kernels and the number of channels for target features, significantly reducing network parameters and computational complexity while maintaining neural network performance.

The biggest achievement of MobileNet is the introduction of deep convolution and point convolution, which reduces the computational complexity of conventional convolution by almost an order of magnitude, becoming the first network to achieve success in the lightweight field. Compared to traditional convolutional neural networks, it greatly reduces model parameters and computational complexity with a small decrease in accuracy.

For traditional conventional convolutions, it is assumed that the input feature is *H* × *W* × *C*. Convolutional kernel size *F* × *F* × *D*. Output feature is *H* × *W* × *D*. The computational complexity O of the overall convolution is

4 $$O = F \times F \times C \times D \times H \times W$$

For MobileNet-DLCNN that has applied deep separable convolution, the overall convolution computation *O’* is

5 $$O^{\prime} = F \times F \times C \times H \times W + C \times D \times H \times W$$

After comparison, it can be seen that

6 $$\frac{{O^{\prime}}}{O} = \frac{F \times F \times C \times H \times W + C \times D \times H \times W}{{F \times F \times C \times D \times H \times W}}{ = }\frac{1}{D} + \frac{1}{{F^{2} }}$$

**Fig. 10:**
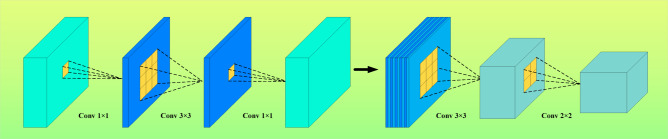
MobileNet-DLCNN model architecture.

It can be seen that MobileNet-DLCNN has significantly reduced computational complexity. The schematic diagram of MobileNet-DLCNN is shown in Fig. 10.

**Fig. 11:**
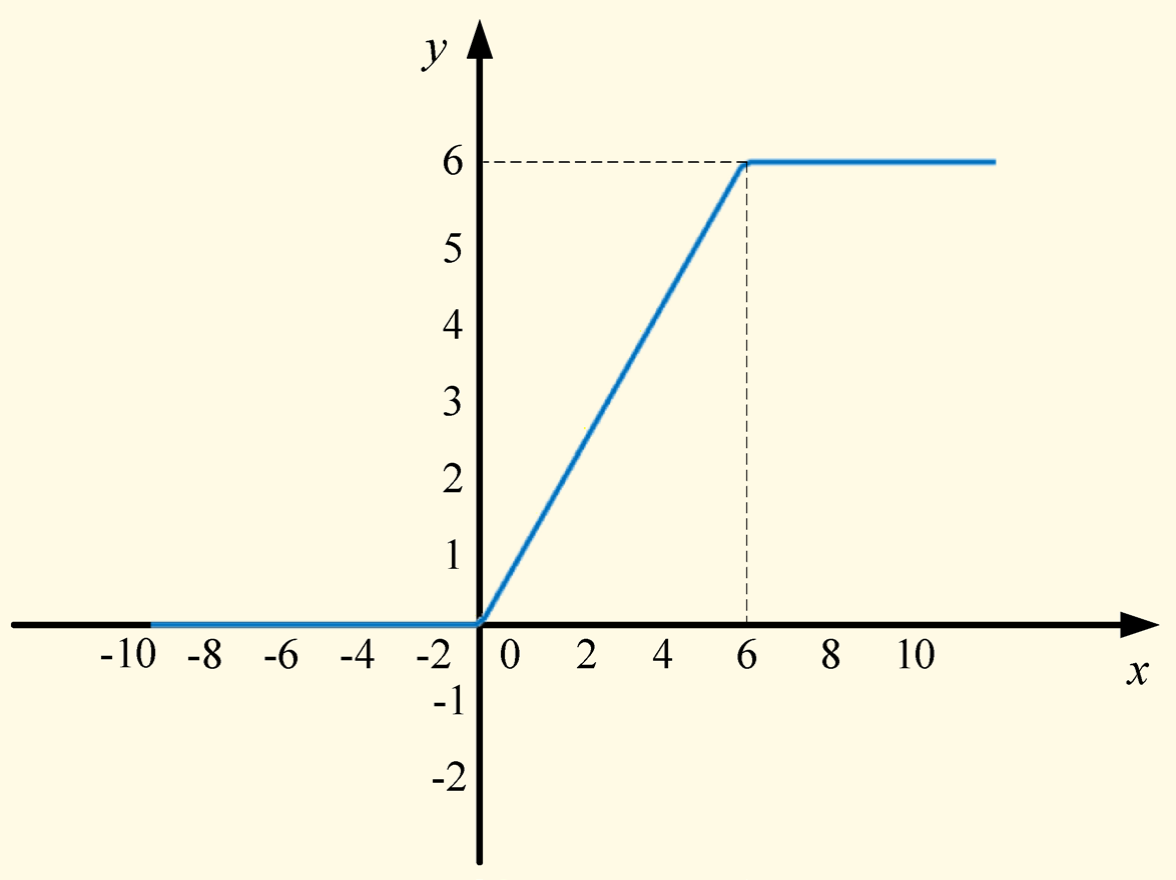
ReLU6 activation function curve.

The activation function used in MobileNet networks is the ReLU6 function, which adds a restriction on the conventional ReLU function, that is, when the input value is greater than 6, the output is 6. This activation function has better robustness at low precision, and its function curve is shown in Fig. 11.

7 $${\mathrm{ReLU}} 6 = \min (\max (0,x),6)$$

The biggest contribution of ShuffleNet is its group operation on the data, which eliminates the need for convolutional computation with each input feature in the convolutional kernel, greatly reducing computational complexity. Each convolutional kernel only needs to calculate the features of its own group, and finally combine the outputs of each group.

**Fig. 12:**
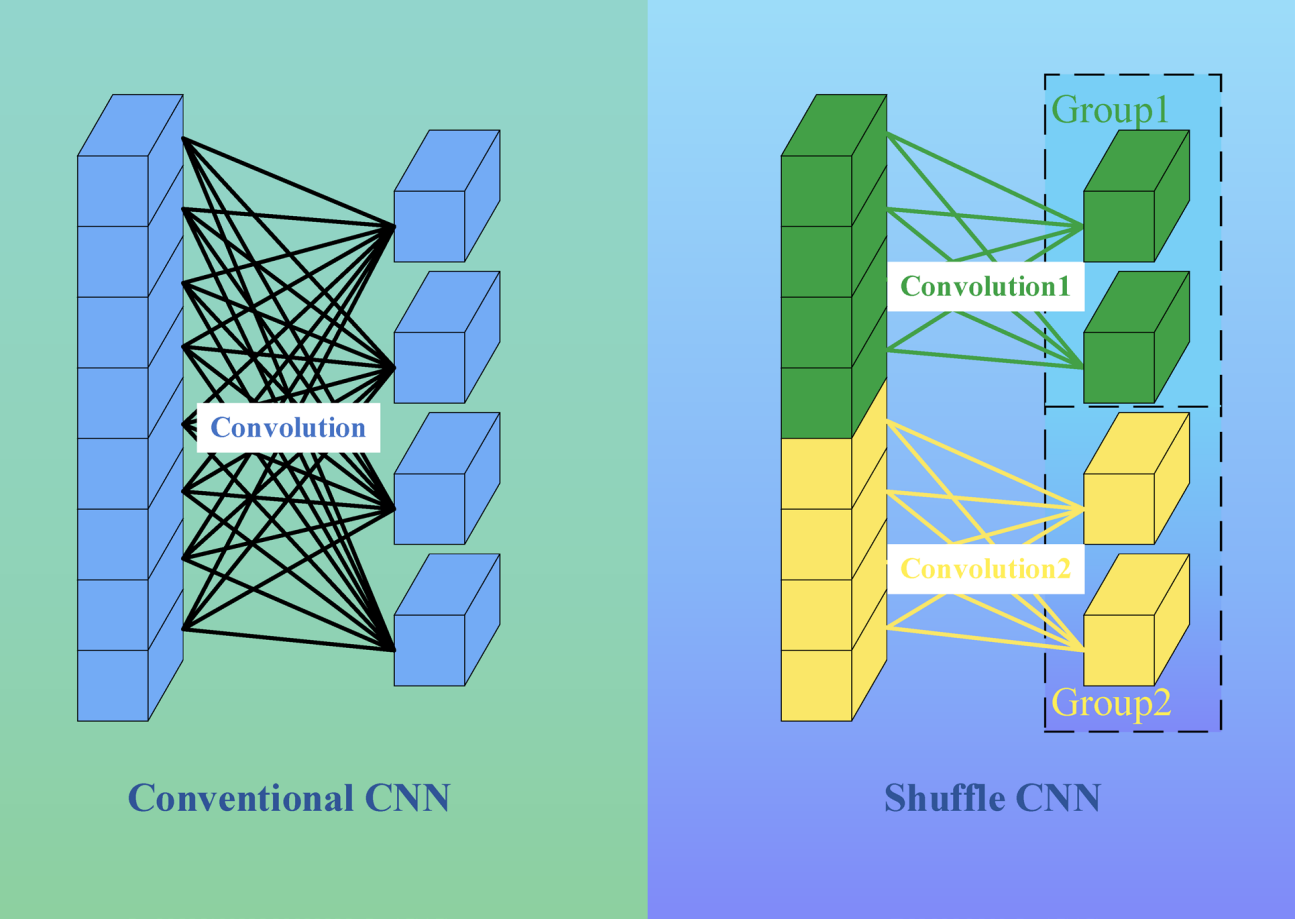
ShuffleNet-DLCNN model architecture.

The ShuffleNet-DLCNN designed in this article combines point by point group convolution with one-dimensional convolution and attention mechanism to form a new lightweight convolutional neural network. This method has high diagnostic efficiency and improves speed at the cost of slightly reducing accuracy. Firstly, batch normalize the standard convolutional blocks to improve the stability of the neural network model. Subsequently, a deeply separable convolution operation is performed, where the number of convolution kernels does not need to be input, only the size of the convolution kernels can be input. Afterwards, channel shuffling and grouping operations are performed, which is the core of lightweight convolution. Finally, combine attention mechanism with one-dimensional convolution. The schematic diagram of ShuffleNet packet convolution is shown in Fig. 12.

The most outstanding contribution of SqueezeNet is its compression processing of neural networks, which utilizes very few parameters but achieves high accuracy. SqueezeNet has a deeper convolutional layer compared to other networks, and often uses small convolutions to increase its nonlinear ability. Leaving aside the advantages and disadvantages of the network itself, the reason why deep networks are so successful is because the cost of reading parameters has shifted to computational complexity. Considering the current state of computer development, the computation time is still less than data access, which is also the root cause of the success of the “multi-level small convolutional kernel” scheme. Therefore, SqueezeNet is also known as an ultra-lightweight network.

**Fig. 13:**
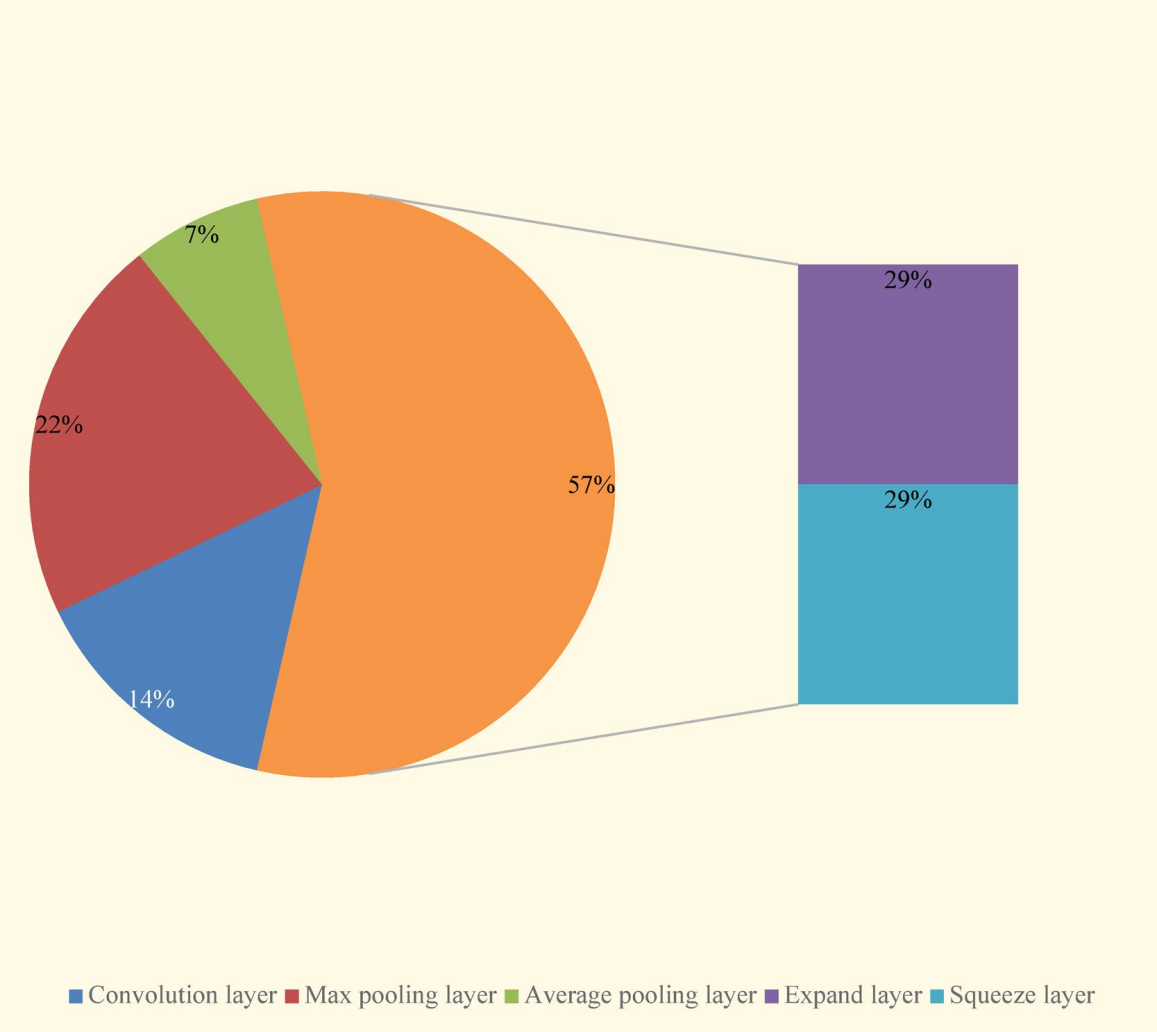
Proportion of parameters in each layer of the SqueezeNet-DLCNN model.

In the SqueezeNet-DLCNN designed in this article, replace the 3 × 3 convolutional kernel with 1 × 1 convolutional kernel and deepening the network layer, increasing the convolutional layer to reduce the number of pooling layers. The Fire Module is the key to SqueezeNet, its core idea is to split each convolutional layer into Squeeze and Expand layers, with an increase in the number of channels after each two modules. Finally, global average pooling was adopted to replace the fully connected layer, solving the problem of limited output size. From Fig. 13, it can be seen that the Fire Module accounts for a large proportion of the entire program and is also a key step in implementing compression operations.

Unlike the general visual backbone design approach adopted by EfficientNet (which focuses on balancing the depth, width, and resolution of the network through composite model scaling) and GhostNet (which focuses on generating redundant feature maps through inexpensive linear transformations), the MobileNet -/ShuffleNet -/SqueezeNet DLCNN model in this study follows a principle of “specialized design for fault diagnosis”. We are not aiming to build a universal image recognition model, but rather based on the classic lightweight idea, using depthwise separable convolution as the core operator, and deeply integrating one-dimensional signal processing characteristics, attention mechanisms, and anti noise training strategies, specifically tailored for extracting robust features from complex industrial vibration signals. Therefore, our model exhibits a better balance of accuracy and efficiency than a general architecture in the noise environment and variable operating conditions of the specific task of bearing fault diagnosis.

### Application of transfer learning in fault diagnosis model

In transfer learning, the source domain and target domain do not need the same data distribution, and the training process only needs less data, which overcomes the shortcomings of traditional machine learning. In this paper, transfer learning is used to diagnose cross working condition transfer fault, and try to train and verify the data under different working conditions and different positions. The deep learning model relies on labeled training data, and in environments without sufficient data labels, training with source data label transmission is required. According to the similarity between the source data label and the target data label, transfer learning fault diagnosis can share the labels of two domains.

**Fig. 14:**
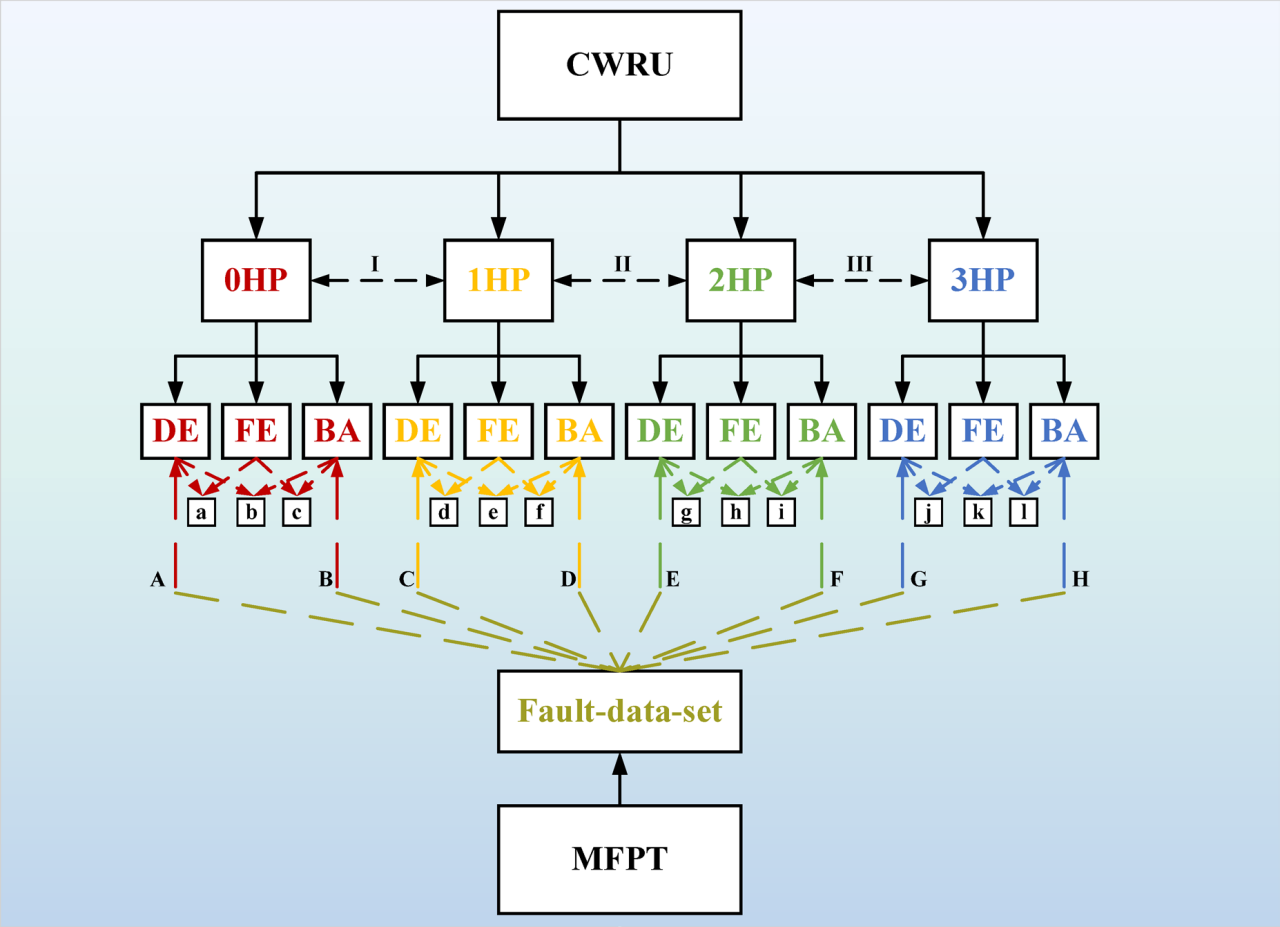
Transfer learning diagnostic process*3.3 Construction of overall model structure.*

In this paper, two bearing failure datasets are used, and colored noise has been added to both bearing failure datasets in order to better explore the anti-jamming ability of the established models. The specific migration learning process is shown in Fig. 14. Transfer learning scenarios are labeled as follows: I ~ III for different working conditions of the CWRU dataset, a ~ l for different signal positions, and A ~ H for transfer learning between the CWRU and MFPT datasets. Specific scenarios corresponding to each letter are detailed in Table 3. The results of transfer learning will be shown in Section “Comparison of migration diagnosis capabilities across different datasets”.

**Table 3:** transfer learning record form.

Scene	Source domain	Target domain	Operating conditions/location description
Ⅰ	CWRU-0HP	CWRU-1HP	Different loads
Ⅱ	CWRU-1HP	CWRU-2HP	
Ⅲ	CWRU-2HP	CWRU-3HP	
a	CWRU-0HP-DE	CWRU-0HP-FE	Different sensor locations
b	CWRU-0HP-DE	CWRU-0HP-BA	
c	CWRU-0HP-FE	CWRU-0HP-BA	
d	CWRU-1HP-DE	CWRU-1HP-FE	Different sensor locations
e	CWRU-1HP-DE	CWRU-1HP-BA	
f	CWRU-1HP-FE	CWRU-1HP-BA	
g	CWRU-2HP-DE	CWRU-2HP-FE	Different sensor locations
h	CWRU-2HP-DE	CWRU-2HP-BA	
i	CWRU-2HP-FE	CWRU-2HP-BA	
j	CWRU-3HP-DE	CWRU-3HP-FE	Different sensor locations
k	CWRU-3HP-DE	CWRU-3HP-BA	
l	CWRU-3HP-FE	CWRU-3HP-BA	
A	MFPT	CWRU-0HP-DE	Cross dataset migration
B	MFPT	CWRU-0HP-BA	
C	MFPT	CWRU-1HP-DE	
D	MFPT	CWRU-1HP-BA	
E	MFPT	CWRU-2HP-DE	
F	MFPT	CWRU-2HP-BA	
G	MFPT	CWRU-3HP-DE	
H	MFPT	CWRU-3HP-BA	

**Fig. 15:**
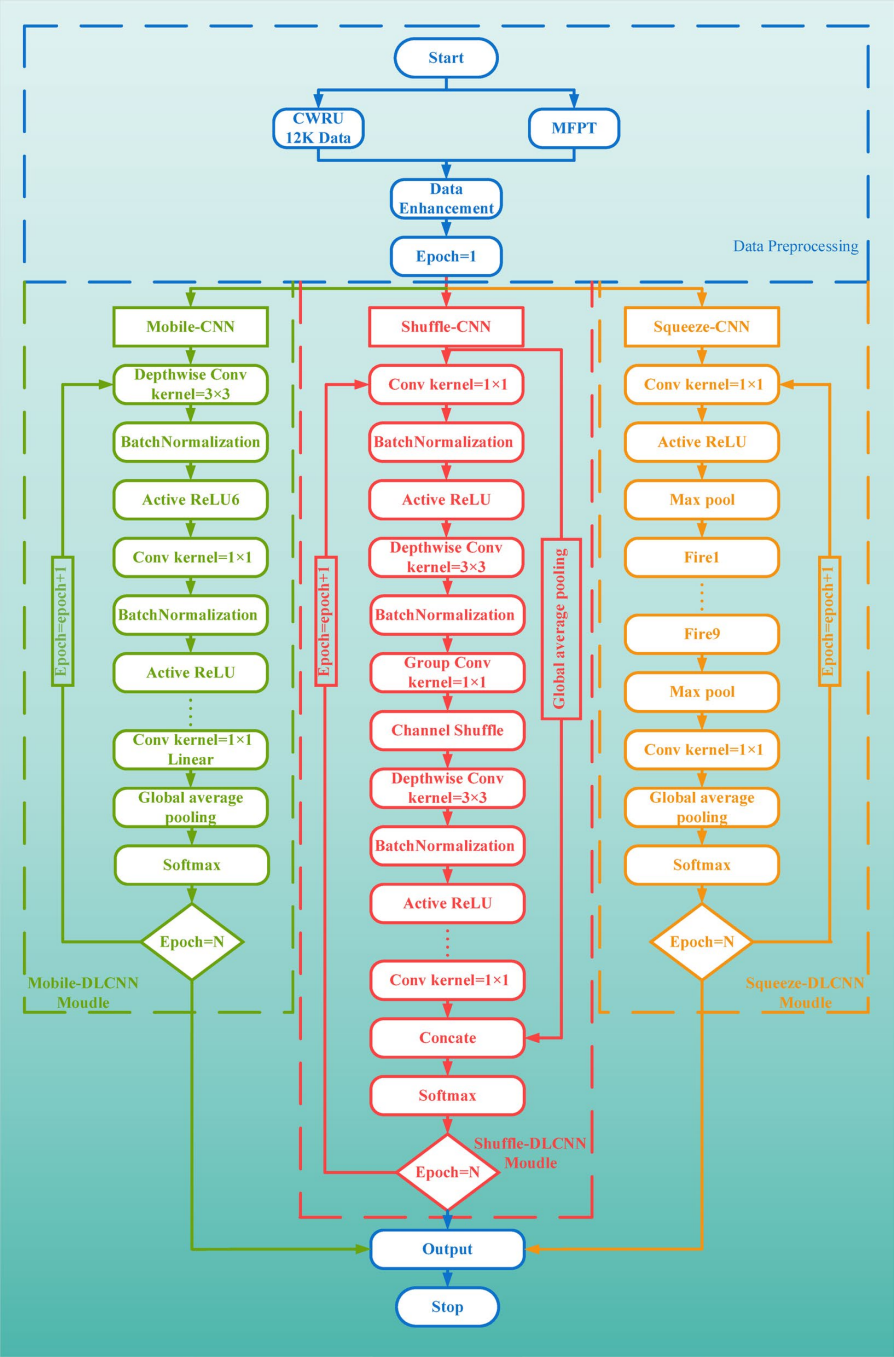
Comparison of the overall process of three lightweight fault diagnosis models.

In this paper, three kinds of anti-noise lightweight convolutional neural networks based on deeply separable convolution are proposed and established, and they are trained by transfer learning. Figure 15 combs the flow charts of the three fault diagnosis models. MobileNet-DLCNN uses the Relu6 activation function, which has better robustness. ShuffleNet-DLCNN performs group obfuscation convolution after performing deep separable convolution, synchronously performing global average pooling, accelerating diagnostic speed. It is also the only diagnostic model among the three that still uses a fully connected layer. SqueezeNet-DLCNN has the deepest convolutional layers and good feature recognition ability.

All three models use an Adam optimizer with a learning rate set to 0.001. The Categorical Crossproperty is used as the loss function. The activation function used by the hidden layer is the ReLU function, the derivative of the ReLU activation function on the positive interval is always 1, so the gradient will not disappear during the back propagation, so it can accelerate the convergence speed of the model. The ReLU function can maximize the screening ability of neurons, and using this function can make calculations fast and the results obtained are very stable. In terms of positive values, ReLU is linear and has better classification performance. The dropout rate is set to 0.2.

## Experiment and discussion

The experiment used in this article will use Python to build a convolutional neural network model in TensorFlow 2.50 environment. TensorFlow is an open-source machine learning framework developed by Google, which can represent computing tasks through data flow graphs and flexibly construct deep neural network models and other machine learning models. The programming software used is PyCharm 2021, which has functions such as intelligent editor, automatic code completion, syntax highlighting, code navigation, debugger, and unit testing tool, enabling developers to develop Python applications more efficiently. The computer CPU used is i5-8250U, with a memory size of 16 GB.

**Fig. 16:**
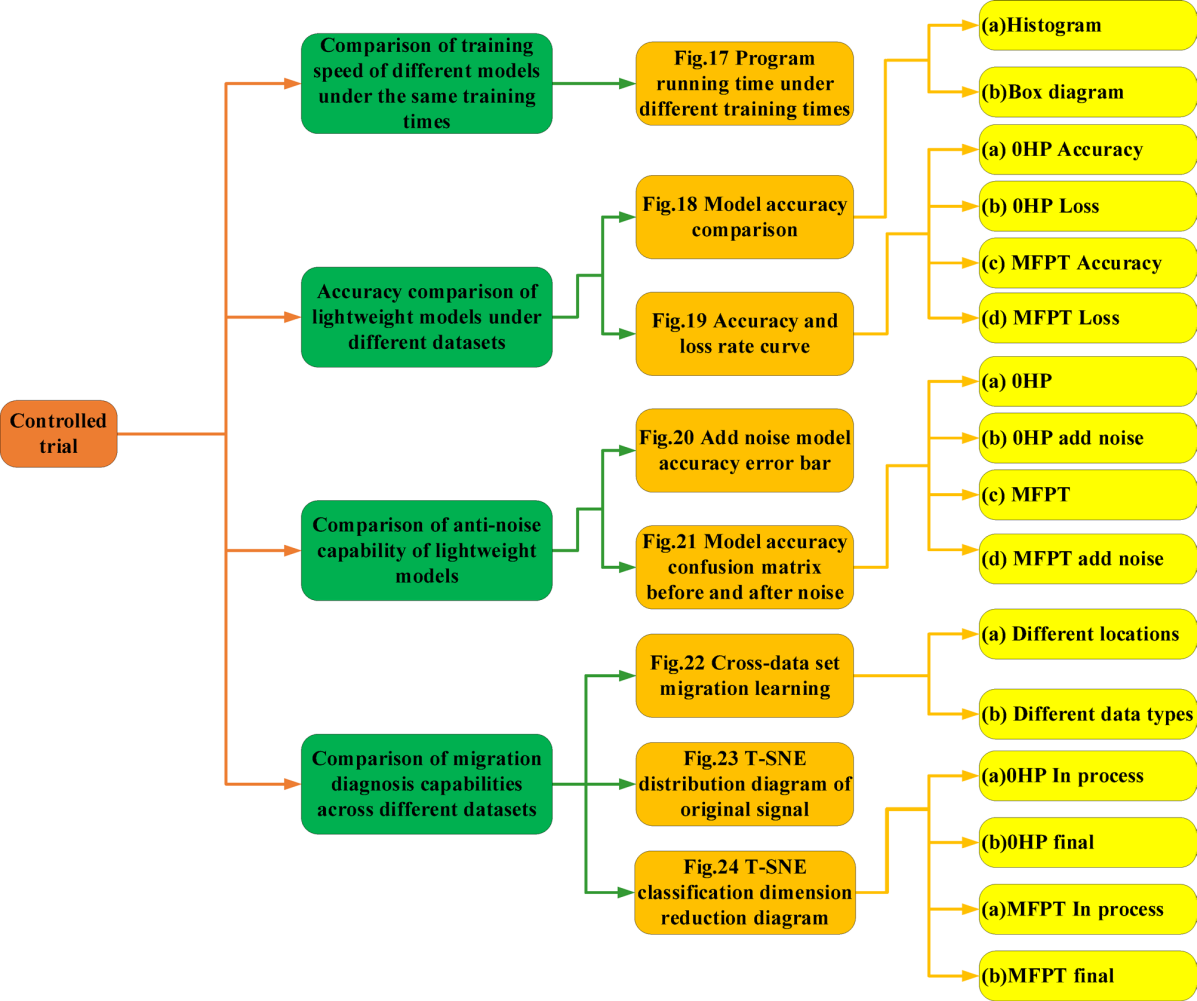
Schematic diagram of controlled trial.

This chapter uses the method of comparative experiments to verify and discuss the various performance of the three lightweight models established, as shown in Fig. 16.

### Comparison of running speeds of different models under the same training times

Although stacking parameters and designing more complex structures can improve the accuracy of models in deep learning, it can also lead to an increase in the computational complexity of the model and a slower training speed^30,31^. Therefore, how well the model predicts is not the only factor to consider, as long training time can lead to an increase in costs. The computation of convolutional neural networks is divided into CPU computation and GPU computation, with GPU computation speed approximately 40% higher than CPU computation speed. Considering that not every computer can be calculated using the GPU method, all subsequent experiments in this article will be conducted using CPU. The network architecture also has a significant impact on the running speed of the model, and the lightweight convolutional neural network model, due to its concise structure, makes the diagnosis speed very fast.

**Fig. 17:**
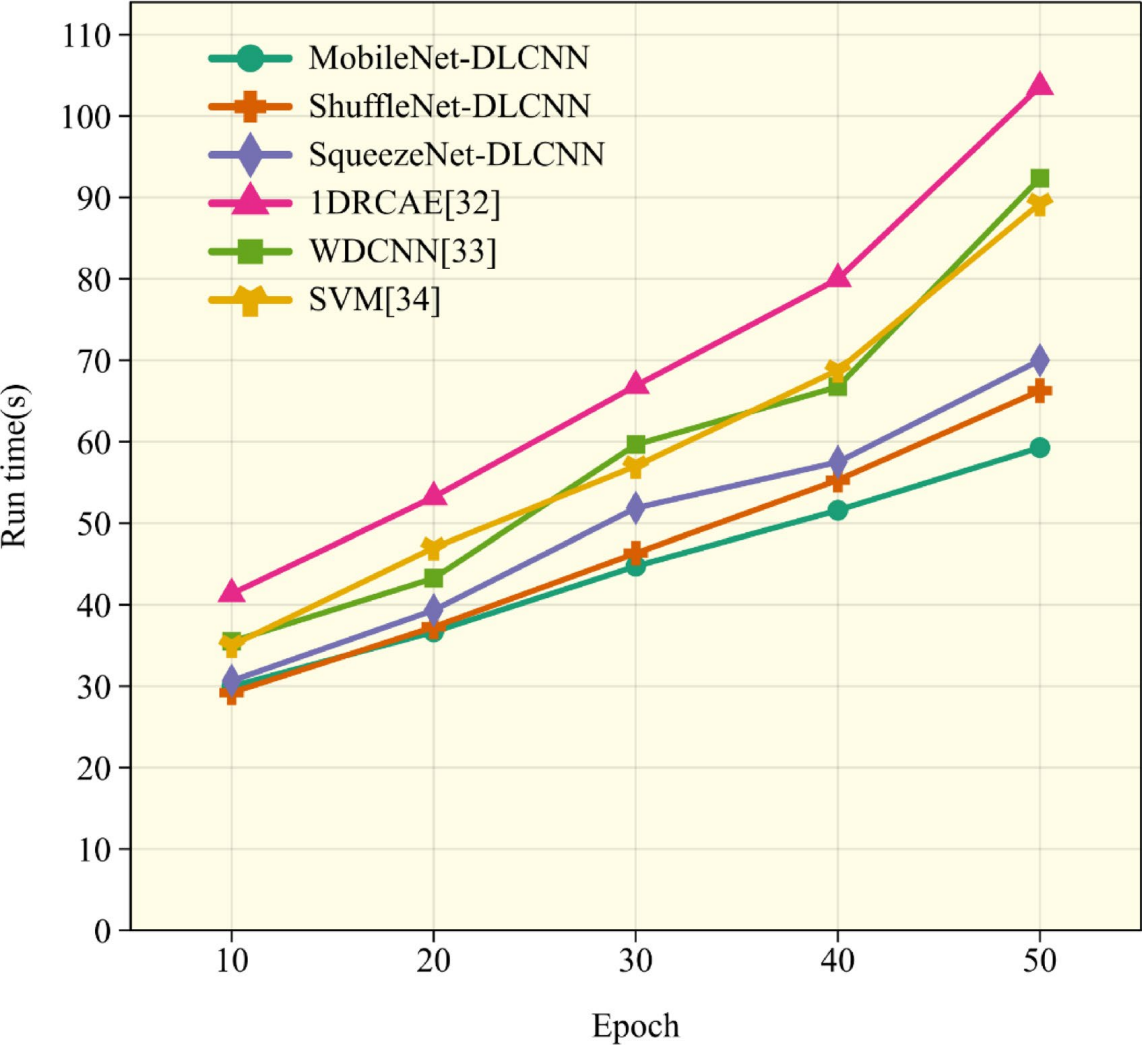
Comparison of program runtime under different training times.

In Fig. 17, the duration of program operation is used to demonstrate the speed of training. In order to demonstrate the fast iteration speed of lightweight convolution, a fault diagnosis model based on One-dimensional Residual Convolutional Autoencoder self-coding(1DRCAE)^32^, wide convolutional kernel neural networks fault diagnosis model(WDCNN)^33^ and Support Vector Machine(SVM)^34^ are added during comparative experiments. From the figure, it can be seen that in the overall comparison, 1DRCAE has the longest diagnostic time due to the presence of an autoencoder, which has the largest computational complexity among all models. Among the three lightweight convolutions established in this article, SqueezeNet-DLCNN takes the longest time due to its deepest convolution layers. However, in overall comparison, it can be seen that it still has a fast diagnostic speed. MobileNet-DLCNN, with its modeling concept of directly reducing parameters in convolution, has the highest diagnostic efficiency, with an average of 50 diagnostic operations completed in one minute.

### Comparison of accuracy of lightweight models under different datasets

Under different operating conditions such as load and speed, the features extracted by signal extraction techniques may vary. In order to verify the generalization characteristics of the three lightweight models in this article and whether the models can operate stably under different operating conditions, four types of fault data of the drive end (DE) under different operating conditions, namely 0HP, 1HP, 2HP, and 3HP, were selected from the Case Western Reserve University dataset, and the dataset compiled from the MFPT bearing dataset of the American Society for Mechanical Fault Prevention and Prevention Technology. Input the fault datasets of both into the model for diagnosis, and the details of the dataset parameters are shown in Table 4.

**Table 4:** Different dataset parameters.

	Train	Valid	Test	Motor load	Speed(r/min)
DT I	7000	2000	1000	0HP	1797
DT II	7000	2000	1000	1HP	1772
DT III	7000	2000	1000	2HP	1750
DT IV	7000	2000	1000	3HP	1730
DT V	7000	2000	1000	MFPT	1500

**Fig. 18:**
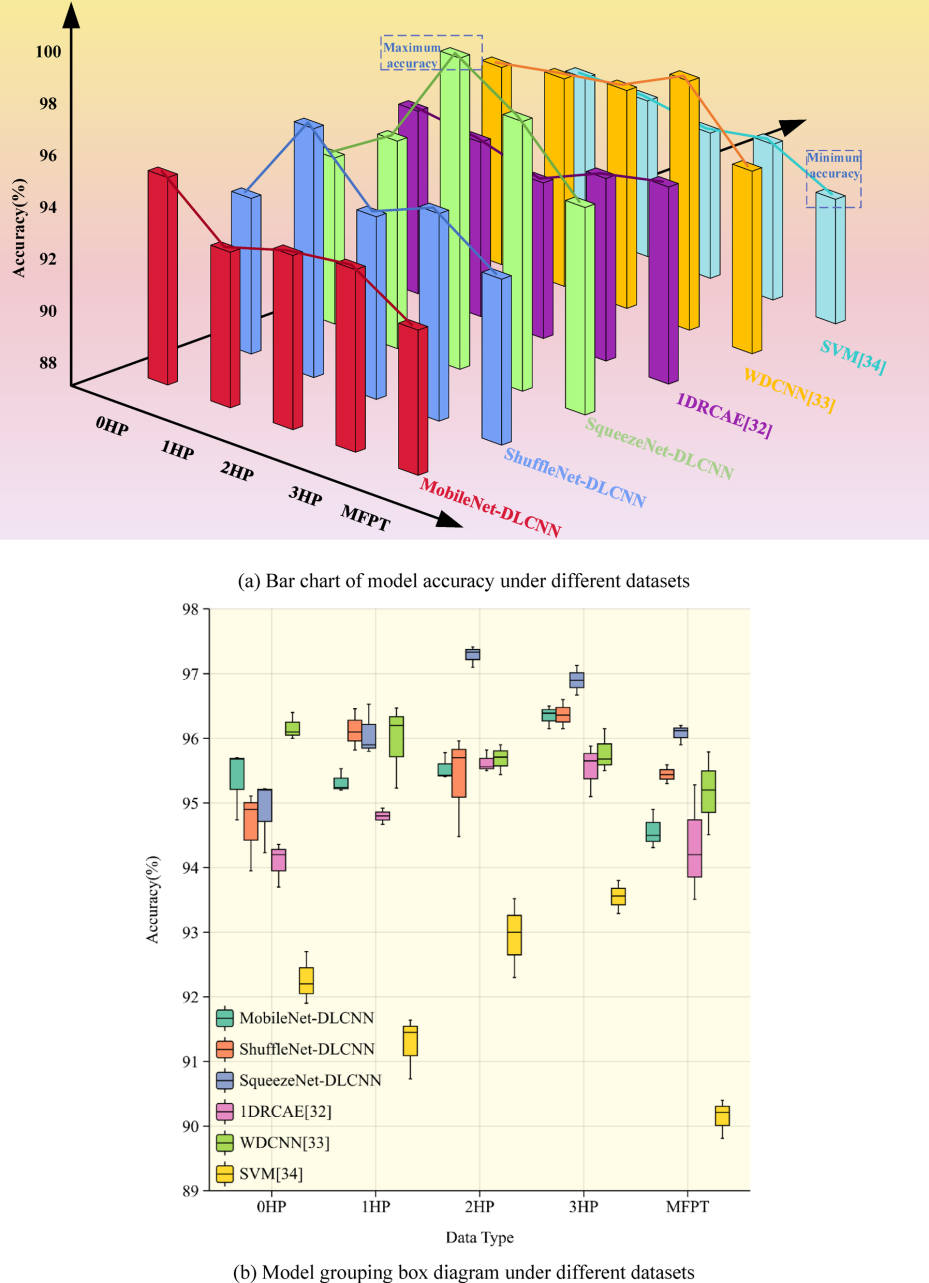
Comparison of model accuracy under different datasets. (**a**) Bar chart of model accuracy under different datasets. (**b**) Model grouping box diagram under different datasets.

The fault data of the driver end (DE) in the Case Western Reserve University dataset is the most stable and is often used to verify the accuracy of various fault diagnosis models. From Fig. 18a, it can be seen that the three lightweight fault diagnosis models have good diagnostic effects, with accuracy rates of over 95%. MobileNet-DLCNN performs well and has high adaptability in the 0HP dataset of CWRU. ShuffleNet-DLCNN has higher accuracy than the other two models at 1HP. SqueezeNet-DLCNN performs well on every dataset, especially on the MFPT dataset. Due to the different data types in the MFPT dataset, the diagnostic accuracy of each model is lower than that of CWRU. SqueezeNet-DLCNN achieved the highest accuracy among the three due to its very deep convolution. Figure 18b shows the grouped box diagrams of the three models constructed in this article under different datasets. Box plots can clearly and intuitively express the distribution range of data, and can show the maximum, minimum, median, quartile, and three-quarter percentile in the graph. The distribution of accuracy of each model can be seen from the figure.

**Fig. 19:**
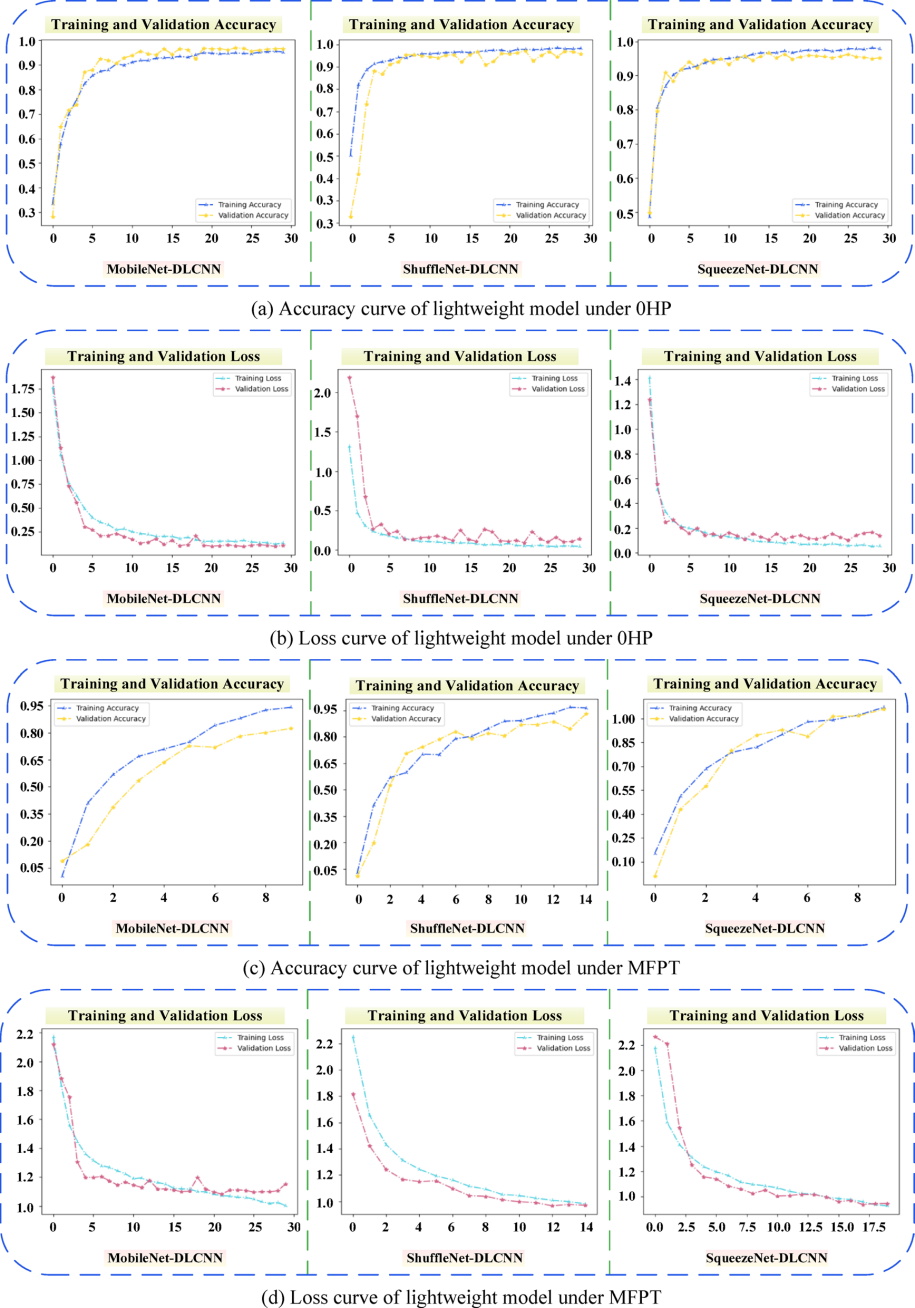
Curve plots of model accuracy and loss rate under different datasets. (**a**) Accuracy curve of lightweight model under 0HP. (**b**) Loss curve of lightweight model under 0HP. (**c**) Accuracy curve of lightweight model under MFPT. (**d**) Loss curve of lightweight model under MFPT.

In order to demonstrate the accuracy of the three lightweight models established in this article more clearly, the accuracy curves and Loss curves of the three models are now output on the 0HP dataset of CWRU and MFPT bearing dataset. As shown in Fig. 19, the ShuffleNet-DLCNN model did not perform well in the early stages of processing, but the fitting effect improved as the diagnosis progressed. When processing the MFPT bearing dataset, SqueezeNet-DLCNN has the best diagnostic performance, and it can be seen from the Loss curve of MobileNet-DLCNN that the fitting effect is slightly worse in the later stage.

### Comparison of noise resistance of lightweight models

Considering the influence of noise in the actual industrial situation, colored noise is added to the data set to simulate the field of bearing application. After the fault signal is converted from time domain to frequency domain, the influence of frequency domain signal on the fault signal is smaller than that of time domain signal. When the data added with colored noise is used, a certain amount of error will be caused.

**Fig. 20:**
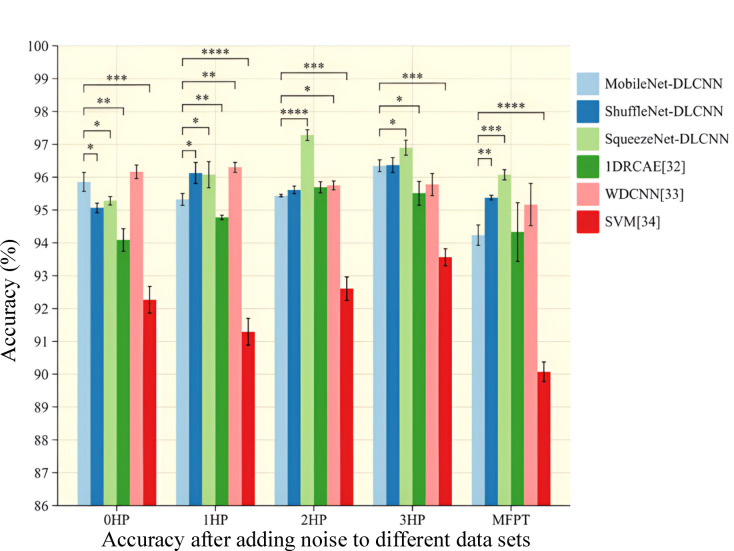
Bar chart of accuracy error bar of noise model added under different datasets.

In order to more intuitively see the change in the accuracy of the model after adding noise, an error bar chart with significant marks is drawn as shown in Fig. 20. Error bar is a graphical representation method used to display the variability or uncertainty of data. Significance can indicate the difference between two columns, with the more asterisks, the more significant the difference. We added WDCNN, which has also undergone anti-noise training, as well as 1DRCAE and SVM, which have not undergone anti-noise training, for comparison. Six models were trained five times in each dataset to calculate the error and standard deviation. From Fig. 20, it can be seen that the accuracy of the model fluctuates after adding noise, and ShuffleNet-DLCNN is the most stable in comprehensive comparison. WDCNN still has high accuracy under noise interference, especially at 0HP, its accuracy and stability are higher than the three lightweight models proposed by the standard. However, in practical experiments, it was found that the single run time of the WDCNN model is longer than that of the lightweight model. After considering all factors, the lightweight convolution proposed in this paper is more excellent. As a machine learning SVM, its diagnostic ability is poorer compared to deep learning, and the addition of noise further affects its diagnostic ability. ShuffleNet-DLCNN demonstrated superior performance in the 1HP dataset, with higher accuracy than the other two models in normal conditions even in the presence of noise interference. When the load changes, the collected bearing vibration signal is unstable. When there are significant changes in speed and load, the impact of noise on the model also increases, making it difficult to extract effective data during convolution processing. Therefore, the performance of the three models in MFPT is poor. As the speed decreases, the impact of noise on SqueezeNet-DLCNN intensifies, resulting in a decrease in diagnostic effectiveness.

**Fig. 21:**
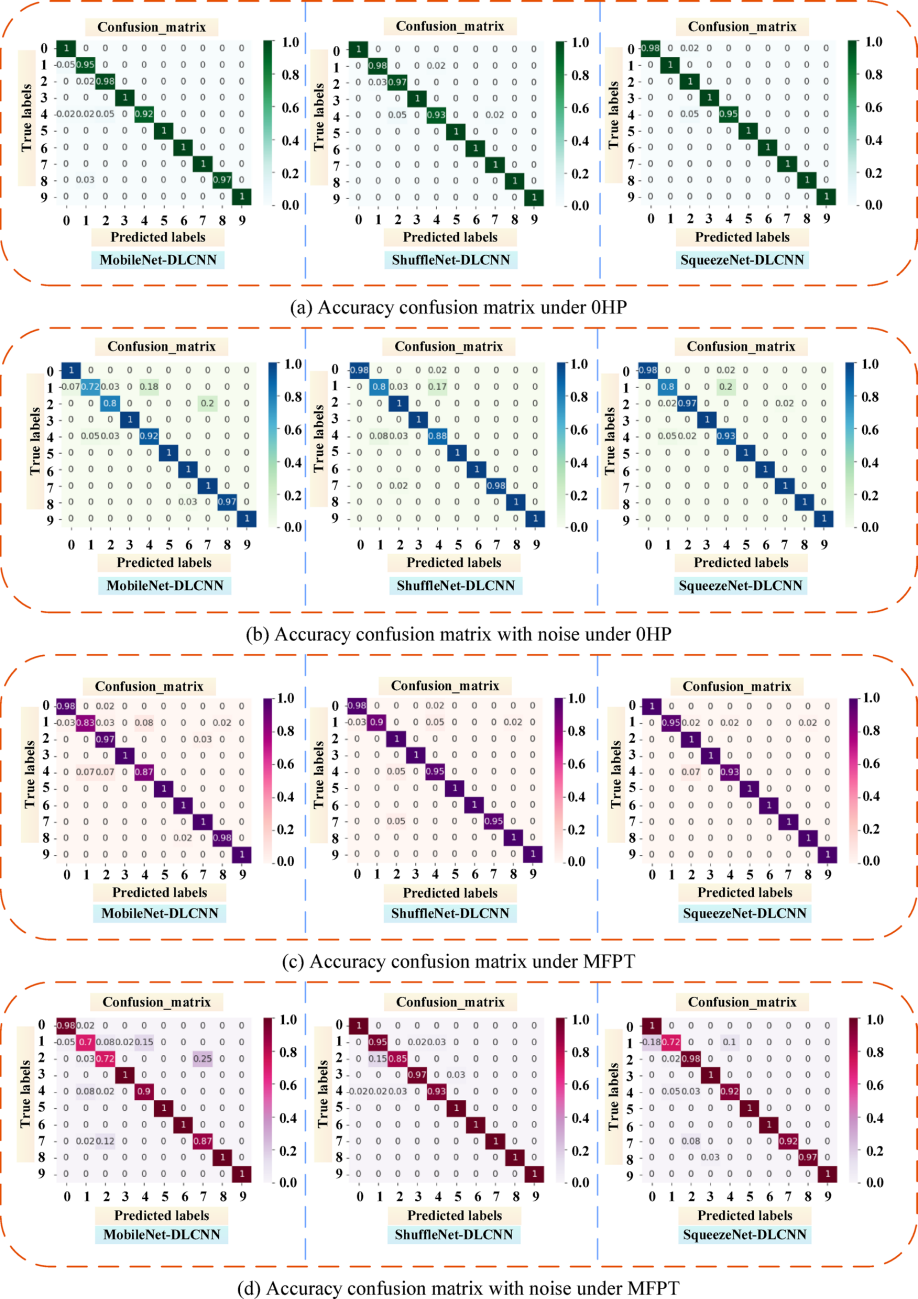
Comparison chart of model accuracy confusion matrix before and after adding noise under different datasets. (**a**) Accuracy confusion matrix under 0HP. (**b**) Accuracy confusion matrix with noise under 0HP. (**c**) Accuracy confusion matrix under MFPT. (**d**) Accuracy confusion matrix with noise under MFPT.

The confusion matrix is an indicator to evaluate the results of the model, which is part of the model evaluation. It is also the most basic, intuitive and simple method to measure the accuracy of the classified model. As shown in Fig. 21, nine types of faults and one normal situation are sorted and classified according to 0–9. The main diagonal of the confusion matrix is the recognition accuracy, the abscissa is the predicted fault type, and the ordinate is the actual fault type. Using the confusion matrix, we can see more clearly the accuracy of the three lightweight models under two data sets and whether noise is added. Figure 21a shows the confusion matrix of the three models under the DE dataset of CWRU 0HP. It can be seen that the three models have poor recognition effect on rolling element faults and good diagnosis effect on outer ring faults. After adding noise, the recognition ability of rolling element faults decreases. As shown in Fig. 21b, the model is prone to misdiagnosing rolling element faults as inner ring faults under noise interference. As shown in Fig. 21c, due to the close proximity of the rolling element to the inner ring, the fault types of the two have a certain degree of overlap. In the MFPT bearing dataset, the model is prone to confuse normal conditions with rolling element faults. After adding noise to the MFPT bearing dataset for fault diagnosis, it was found that the diagnostic ability of MobileNet-DLCNN decreased most significantly, and for the first time, there was confusion between rolling element faults and outer ring faults, as shown in Fig. 21d.

### Comparison of migration diagnosis capabilities across different datasets

Under different loads, the number of characteristics of vibration signals varies, the amplitude is not constant, and the period and phase of oscillation also vary greatly. This will result in the classifier being unable to correctly classify faults of the same type under different loads, thereby reducing the recognition speed of the diagnostic system.

As shown in Fig. 14, the data set of CWRU can be divided into 0HP, 1HP, 2HP and 3HP according to different rotational speeds, and the transfer learning between them is recorded as I ~ III. Because the vibration signals are collected at different positions, the signals collected at three different positions under each data set are transfer learning, and the process is recorded as a ~ l. Considering that the MFPT dataset and the CWRU dataset belong to different data types, the transfer learning between the two datasets is recorded as A ~ H. In the training process of transfer learning, one party’s data is used as the training set and verification set, and the other party’s data is used as the test set. Using the Adam gradient descent algorithm to adaptively optimize the parameters, the epoch is selected as 50. After multiple experiments, the batch size was determined to be 64. As can be seen from Table 5, the accuracy of ShuffleNet-DLCNN and SqueezeNet-DLCNN increases gradually with the gradual decrease of rotational speed during transfer learning between data sets at different rotational speeds. MobileNet-DLCNN has weak diagnostic ability under variable speed transfer learning, and is not easy to adapt to speed changes in actual work.

**Table 5:** Accuracy of transfer learning between data sets with different rotational speeds.

	MobileNet-DLCNN (%)	ShuffleNet-DLCNN (%)	SqueezeNet-DLCNN (%)
I	94.4	94.5	93.9
II	92.1	95.3	96.1
III	93.6	95.7	96.4

**Fig. 22:**
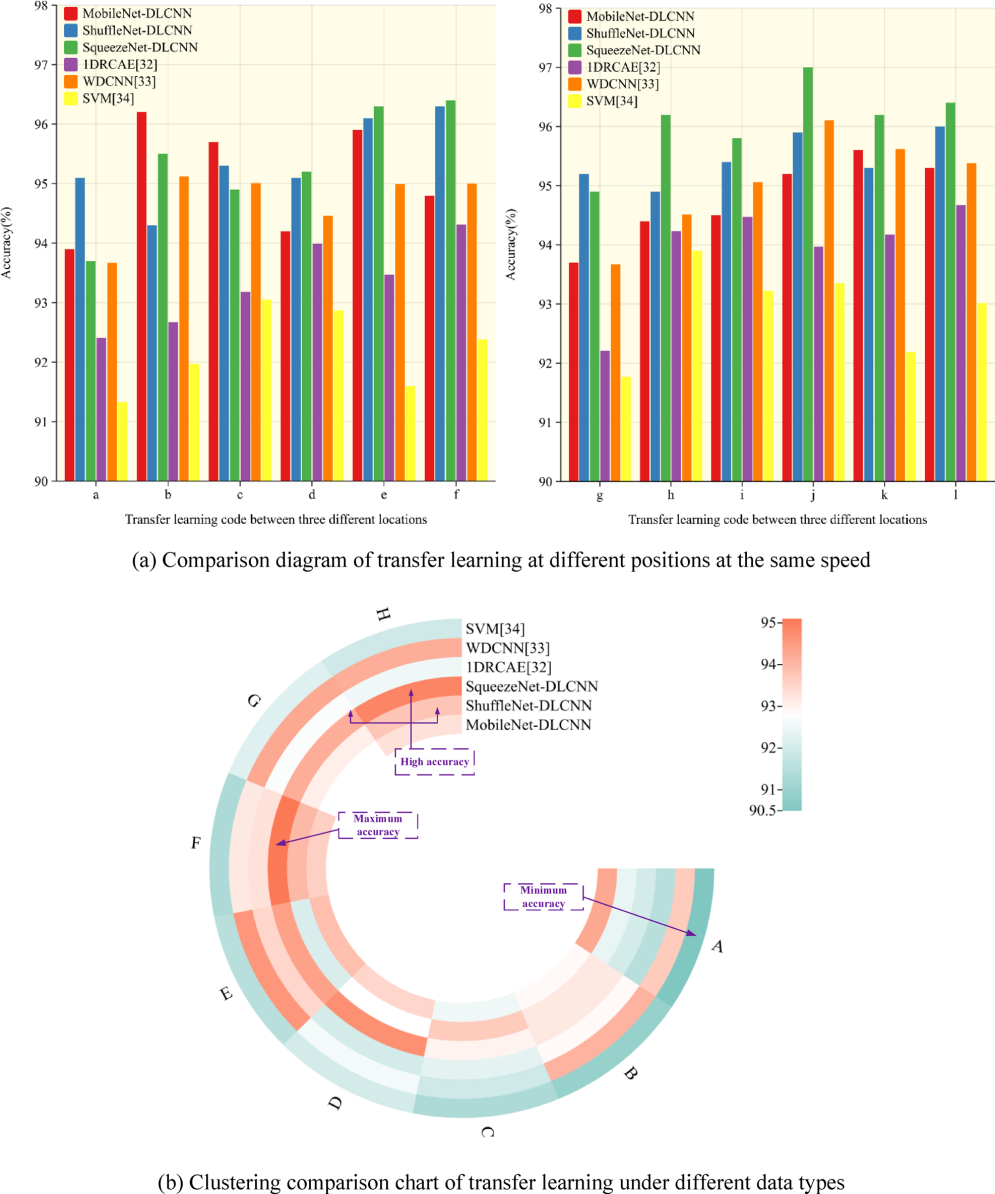
Cross dataset transfers learning accuracy image. (**a**) Comparison diagram of transfer learning at different positions at the same speed. (**b**) Clustering comparison chart of transfer learning under different data types.

Figure 22 shows the comparison of transfer learning across datasets. According to Fig. 22a, at the same speed, there is a certain rule of transfer learning between different signal acquisition positions. Generally speaking, the migration diagnosis ability between the drive end (DE) and the fan end (FE) is weak, which may be due to the loss of vibration signals during transmission. The diagnostic effect is relatively stable when it comes to the base end (BA). On the whole, SqueezeNet-DLCNN still has the best diagnostic ability, and the average accuracy of transfer learning among bearing data sets of Case Western Reserve University is higher than 95%. Figure 22b shows the transfer learning under different data types. Here, the data of drive end (DE) and base end (BA) under MFPT dataset and Case Western Reserve University data sets under four different working conditions are used for transfer learning. In order to make the comparison more obvious, WDCNN,1DRCAE and SVM are added here.

It can be seen from Table 6 that MobileNet-DLCNN has the best learning effect under the learning of DE data and MFPT data migration under 0HP working condition, and the results of Fig. 20 also show that it has the strongest anti-noise ability under 0HP. From this, it can be concluded that MobileNet-DLCNN has the optimal diagnostic capability at 0HP. In transfer learning other than those involving 0HP, ShuffleNet-DLCNN and SqueezeNet-DLCNN have similar trends, and SqueezeNet-DLCNN has a higher diagnostic ability. Even on two datasets with different data types, the average accuracy of SqueezeNet-DLCNN reached 94%. The diagnostic performance of WDCNN is still excellent at 0HP, but its diagnostic ability shows a cliff like decline at 1HP, and the subsequent diagnostic trend is also different from that of lightweight convolution. It is speculated that this situation may be due to the different ideas of WDCNN and the three lightweight convolutional variable condition diagnosis proposed in this article. WDCNN adopts a wide convolutional kernel, which can collect more data at a single time. The three lightweight convolutions proposed in this article use compression, grouping, small convolutional kernels, and deepening the network layers for diagnosis. 1DRCAE is also capable of transfer learning due to the residual network and self-coding nonlinear dimensionality reduction. Due to the lack of consideration for variable operating conditions during the design of SVM, the overall diagnostic performance is poor.

**Table 6:** Accuracy of transfer learning under different data types.

	MobileNet-DLCNN	ShuffleNet-DLCNN (%)	SqueezeNet-DLCNN (%)	1DRCAE^32^ (%)	WDCNN^33^ (%)	SVM^34^ (%)
A	94.3%	92.4	92.0	91.5	93.7	90.5
B	92.9%	93.2	93.2	92.9	94.1	90.9
C	92.5%	93.7	93.0	92.3	91.9	91.3
D	93.5%	92.8	94.7	92.0	92.6	92.0
E	93.9%	92.1	94.4	93.5	94.6	91.5
F	93.6%	94.0	95.1	93.3	93.2	91.3
G	92.8%	93.1	94.2	92.7	94.3	92.2
H	93.3%	93.8	94.9	92.5	94.2	91.9

**Fig. 23:**
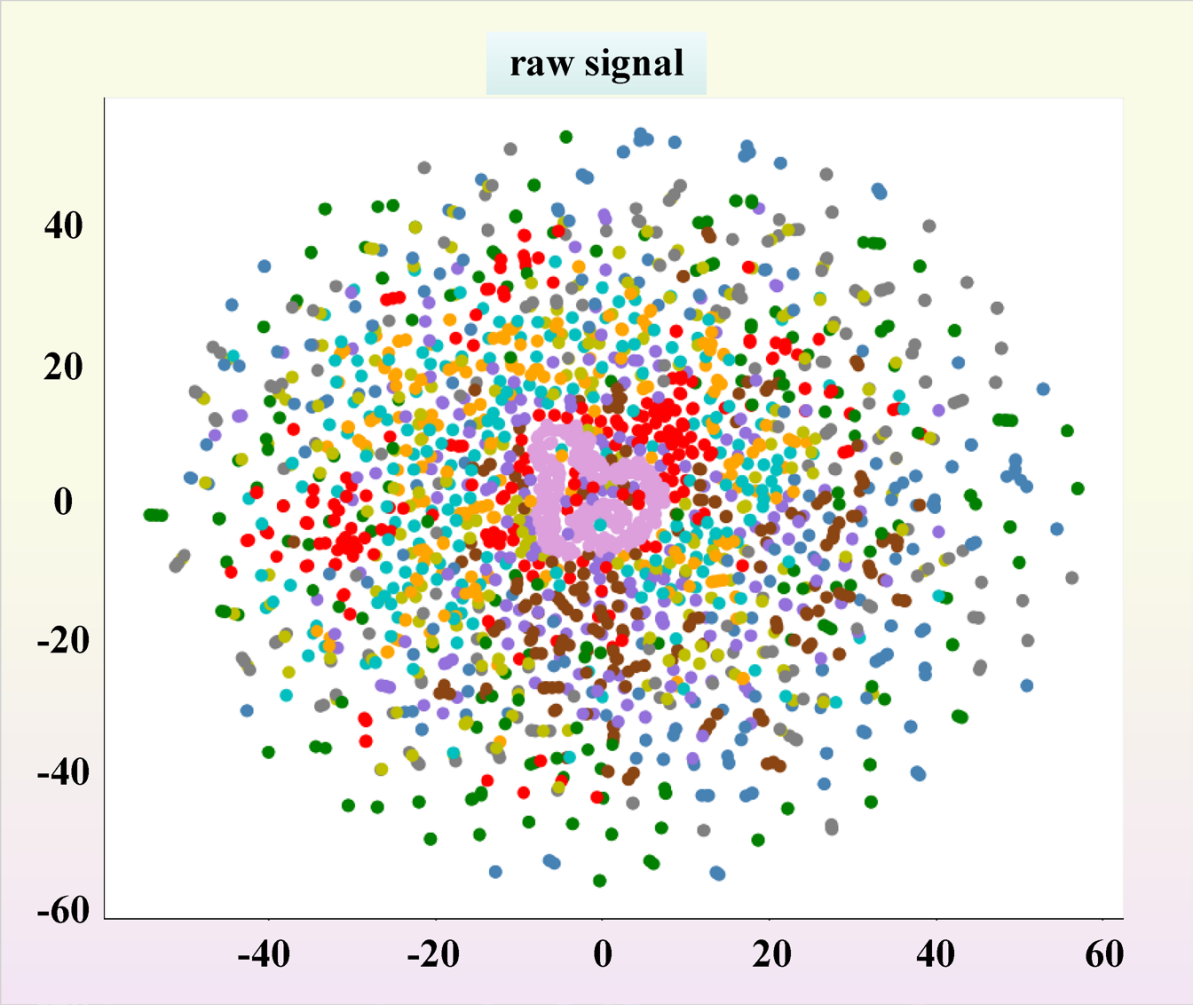
T-SNE distribution map of original signal.

In order to have a clearer view of the diagnostic capabilities of the three models for bearing faults, dimensionality reduction operations were performed on the three models and images were output. The so-called dimensionality reduction refers to using 2 or 3 dimensions to represent multidimensional data. The full name of T-SNE is T-distributed Stochastic Neighbor Embedding. T-distribution random nearest neighbor embedding is a technology that combines dimensionality reduction and rendering. It is based on SNE visualization enhancement and solves the characteristics of sample distribution congestion and SNE boundary blurring after imaging. T-SNE models the similarity of the original space as probability density, and the distribution of similarity is given by Gaussian distribution. The original signal graph without classification operation after dimensionality reduction is shown in Fig. 23. In short, in the original space, the similarity between a point and other points can be expressed by a probability density distribution:

8 $$p_{j|i} = \frac{{\exp ( - \frac{{\left\| {xi - xj} \right\|^{2} }}{{2\sigma_{i}^{2} }})}}{{\Sigma_{k \ne i} \exp ( - \frac{{\left\| {xi - xk} \right\|^{2} }}{{2\sigma_{i}^{2} }})}}$$

In the dimensionality reduced space, we use the student t-distribution instead of the Gaussian distribution because the student t-distribution can retain more similarity over longer distances. So in the target space after dimensionality reduction, the joint probability distribution is:

9 $$q_{j|i} = \frac{{(1 + \left\| {yi - yj} \right\|^{2} )^{ - 1} }}{{\Sigma_{k \ne l} (1 + \left\| {yk - yl} \right\|^{2} )^{ - 1} }}$$

**Fig. 24:**
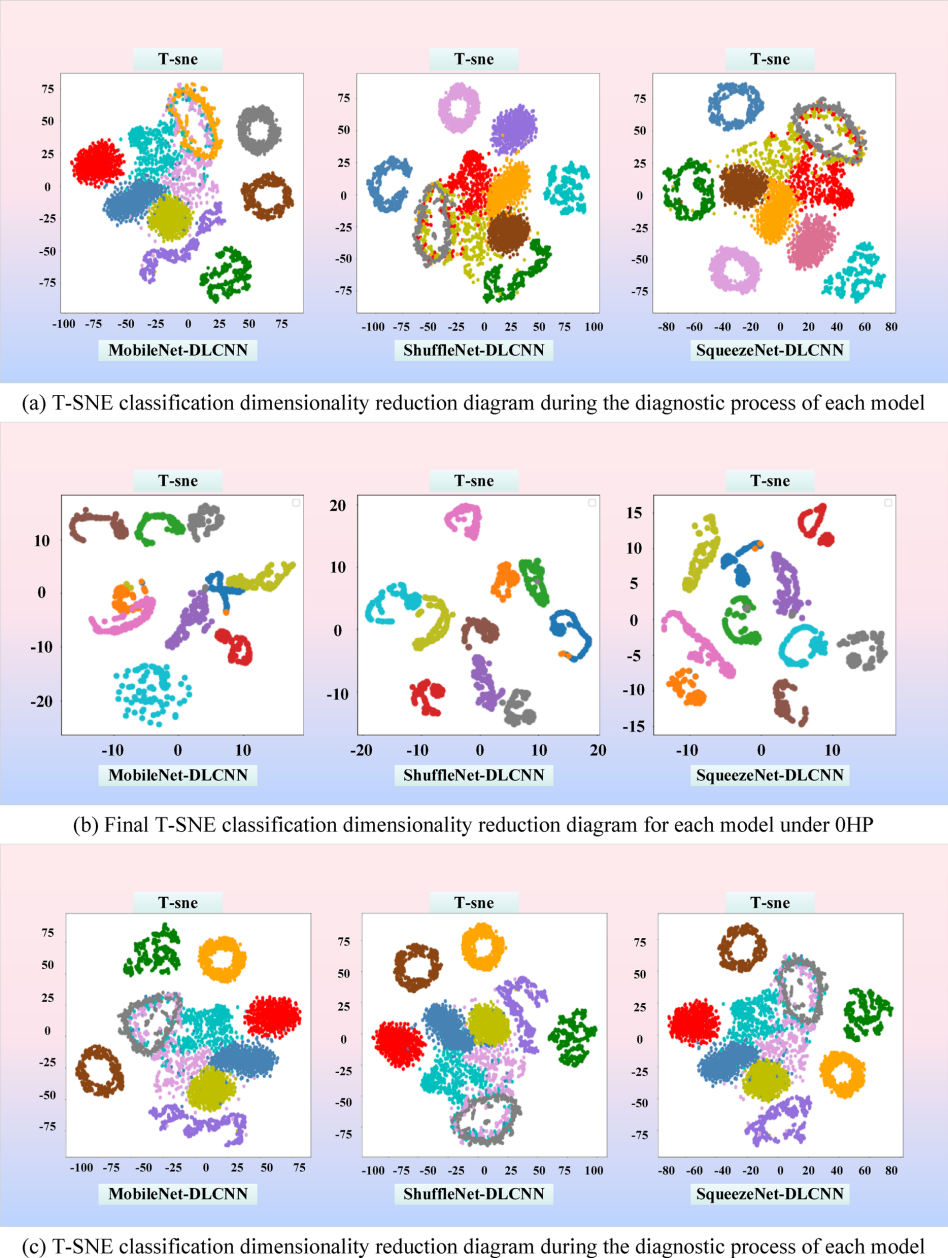

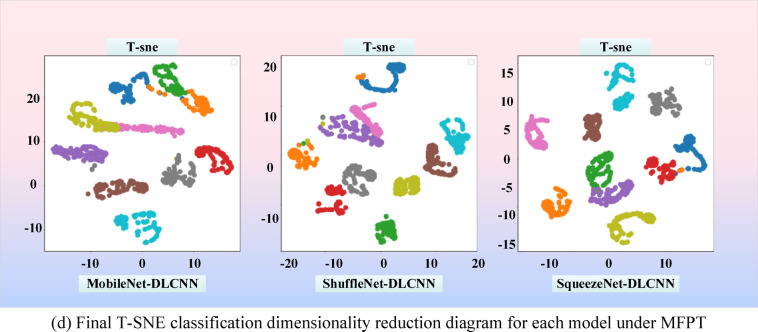
T-SNE classification and dimensionality reduction diagram for each dataset. (**a**) T-SNE classification dimensionality reduction diagram during the diagnostic process of each model under 0HP. (**b**) Final T-SNE classification dimensionality reduction diagram for each model under 0HP. (**c**) T-SNE classification dimensionality reduction diagram during the diagnostic process of each model under MFPT. (**d**) Final T-SNE classification dimensionality reduction diagram for each model under MFPT.

From Fig. 23, it can be seen that the original input signal has the maximum entropy and the highest degree of confusion. Various faults are mixed together and cannot be separated, and the feature interval is relatively fuzzy. During the diagnostic process, a certain moment is intercepted for dimensionality reduction, as shown in Fig. 24a. It can be seen that all three models exhibit certain classification capabilities after undergoing a period of convolution operation. Fault data of the same category has begun to converge, and some fault data has even been completely separated.

It can be seen that the classification ideas of ShuffleNet-DLCNN and SqueezeNet-DLCNN are similar, and their separation situation is similar. The final classification and dimensionality reduction diagrams of the three models are shown in Fig. 24b. As shown in the figure, the fault data is separated into its own groups, and many fault data of the same type have already overlapped. There is another type of failure in MobileNet-DLCNN that is not fully clustered, and there are entanglements between several groups. Compared with the other two lightweight models, the classification performance is worse, with all 10 types clustered and more dispersed.

To quantitatively evaluate the clustering quality, we calculated the contour scores for each model. SqueezeNet DLCNN achieved the highest score (0.82 under CWRU and 0.75 under MFPT), indicating clearer differentiation between fault categories. In contrast, SqueezeNet DLCNN has a more significant classification performance, minimal diagnostic error, and no inter group interference. In Figs. 24c and d, it can be observed that the classification performance of the MFPT dataset is slightly inferior to that of the CWRU dataset. The reason for this situation may be due to different fault classification situations.

The lightweight, efficient, and noise resistant deep learning framework designed by our research institute has potential applications beyond mechanical fault diagnosis. The core advantage of this framework—extracting robust features from complex signals and performing efficient classification—makes it highly suitable for other fields with similar challenges. For example, in the field of medicine, understanding the crosstalk between lung cancer pleural metastases and tumor epithelial cells relies on the analysis of complex cell communication signals ^35^; In environmental acoustic monitoring, it can be used for species call recognition or abnormal event detection. Recent research in non mechanical fields, such as disease prediction models based on deep learning, has also demonstrated the generality of similar techniques ^36^. Therefore, transferring this model to these fields is a highly promising future direction.

## Conclusions

Aiming at the challenges of long time-consumption, sensitivity to noise interference, and difficulty in adapting to variable working conditions faced in the field of bearing fault diagnosis, this study explores a lightweight and strong noise-resistant convolutional neural network model, aiming to improve the real-time, accuracy, and environmental adaptability of bearing fault diagnosis. Specifically, this study achieved the following main results:

1. In this study, three lightweight neural network models based on depth-separable convolution-MobileNet-DLCNN, ShuffleNet-DLCNN, and SqueezeNet-DLCNN-are innovatively designed and implemented for bearing fault diagnosis. These models effectively reduce the number of model parameters and computational complexity by introducing the depth-separable convolution technique, thus significantly reducing the computation time and significantly improving the processing efficiency while maintaining the high-precision diagnostic performance. This design strategy not only optimizes the resource utilization efficiency, but also provides strong support for the development of real-time fault diagnosis system.
2. In view of the complexity and diversity of noise signals in real industrial environments, this study creatively introduces colored noise components on the basis of the traditional dataset to simulate a test environment closer to real working conditions. By training on these datasets containing color noise, the lightweight models proposed in this paper demonstrate excellent resistance to noise interference. In particular, experimental results on standard datasets such as CWRU and MFPT show that these models not only maintain the original fast diagnosis advantage, but also significantly enhance the stability and accuracy in complex noise environments.
3. In order to verify the generalization ability of the model and its adaptability to different operating conditions, this study further employs a transfer learning technique to apply the trained model to tests under the same operating conditions, different operating conditions, and a completely new dataset. The experimental results show that the MobileNet-DLCNN, ShuffleNet-DLCNN and SqueezeNet-DLCNN models all exhibit good migration learning and can maintain high diagnostic accuracy under different conditions, proving their strong generalization ability and potential for practical applications.
4. Comprehensive performance analysis and comparison comprehensive experimental test results show that the three lightweight anti-colored noise convolutional neural network models constructed in this paper exhibit high efficiency, high accuracy, strong generalization and excellent noise resistance in bearing fault diagnosis. Compared with traditional methods, these models achieve significant improvement in several key performance indicators, providing new ideas and solutions for the technical progress in the field of bearing fault diagnosis.

Research Limitations and Future Work.

Although this study has achieved the expected results, there are still some limitations that need to be further explored in future work:

1. Limitations of data types: The validation of the current model is only based on vibration signal datasets (CWRU and MFPT), and its diagnostic effectiveness for other types of industrial signals (such as acoustic signals, current signals, etc.) is not yet known.
2. The depth of model comparison: The comparison with the latest and most advanced lightweight architectures (such as EfficientNetV2, GhostNet, etc.) is not sufficient, and more comprehensive performance benchmark testing is needed in the future.
3. Real time deployment verification: the current research is completed in the experimental environment, and the final value of the model needs to be further verified through actual deployment and online diagnosis on embedded devices or edge computing nodes.

In response to the above limitations, future research will focus on: (1) exploring the application of models in multi-sensor information fusion fault diagnosis; (2) Deeply compare and integrate the proposed architecture with more cutting-edge lightweight models; (3) Promote the embedded deployment of models in real industrial scenarios, achieving a leap from “algorithmic effectiveness” to “practical implementation”.

## Data Availability

The datasets used and/or analysed during the current study available from the corresponding author on reasonable request.
